# Frustration can Limit the Adaptation of Promiscuous Enzymes Through Gene Duplication and Specialisation

**DOI:** 10.1007/s00239-024-10161-4

**Published:** 2024-03-12

**Authors:** Michael Schmutzer, Pouria Dasmeh, Andreas Wagner

**Affiliations:** 1 Department of Evolutionary Biology and Environmental Studies, University of Zurich, Zurich, Switzerland; 2 Swiss Institute of Bioinformatics, Lausanne, Switzerland; 3 Center for Human Genetics, Philipps University of Marburg, Marburg, Germany; 4 Santa Fe Institute, Santa Fe, NM USA

**Keywords:** Enzyme promiscuity, Evolution, Gene duplication, Enzyme kinetics, Evolutionary biophysics

## Abstract

Virtually all enzymes catalyse more than one reaction, a phenomenon known as enzyme promiscuity. It is unclear whether promiscuous enzymes are more often generalists that catalyse multiple reactions at similar rates or specialists that catalyse one reaction much more efficiently than other reactions. In addition, the factors that shape whether an enzyme evolves to be a generalist or a specialist are poorly understood. To address these questions, we follow a three-pronged approach. First, we examine the distribution of promiscuity in empirical enzymes reported in the BRENDA database. We find that the promiscuity distribution of empirical enzymes is bimodal. In other words, a large fraction of promiscuous enzymes are either generalists or specialists, with few intermediates. Second, we demonstrate that enzyme biophysics is not sufficient to explain this bimodal distribution. Third, we devise a constraint-based model of promiscuous enzymes undergoing duplication and facing selection pressures favouring subfunctionalization. The model posits the existence of constraints between the catalytic efficiencies of an enzyme for different reactions and is inspired by empirical case studies. The promiscuity distribution predicted by our constraint-based model is consistent with the empirical bimodal distribution. Our results suggest that subfunctionalization is possible and beneficial only in certain enzymes. Furthermore, the model predicts that conflicting constraints and selection pressures can cause promiscuous enzymes to enter a ‘frustrated’ state, in which competing interactions limit the specialisation of enzymes. We find that frustration can be both a driver and an inhibitor of enzyme evolution by duplication and subfunctionalization. In addition, our model predicts that frustration becomes more likely as enzymes catalyse more reactions, implying that natural selection may prefer catalytically simple enzymes. In sum, our results suggest that frustration may play an important role in enzyme evolution.

**Supplementary Information:**

The online version contains supplementary material available at 10.1007/s00239-024-10161-4.

## Introduction

Virtually all enzymes catalyse more than one chemical reaction, a phenomenon that is called enzyme promiscuity (Glasner et al. 2020; Peracchi 2018; Khersonsky and Tawfik 2010; Nobeli et al. 2009; Copley 2017). Some promiscuous enzymes are heavily specialised towards catalysing a single reaction with one substrate (Tawfik and Gruic-Sovulj 2020) but also catalyse side reactions at low and physiologically irrelevant rates (Copley 2017; Khersonsky and Tawfik 2010). Current estimates suggest that enzymes catalyse on average 10 side reactions (Copley 2017), although evidence suggests the number of reactions per enzyme could be substantially higher (Huang et al. 2015). Other promiscuous enzymes are true generalists. These generalist enzymes catalyse reactions with multiple substrates at comparable rates. Some of these reactions may be quite different from each other (Copley 2017). Most enzymes are far less discriminating between substrates than they could be (Peracchi 2018). For example, the repair enzymes that detoxify the metabolic waste of central carbon metabolism are often generalists (Bommer et al. 2020; Zhang et al. 2012). We do not know whether and when evolution favours specialist or generalist enzymes.

In this study, we will use the general term ‘activity’ to refer to the ability of an enzyme to catalyse either a particular kind of reaction, or a reaction with a given substrate. The extent of this ability is commonly quantified in terms of catalytic efficiency (Eisenthal et al. 2007). We will refer to activities favoured and preserved by selection as functional activities (Keeling et al. 2019). If an enzyme has multiple such activities, we consider it multifunctional. Because we work with a dataset in which it is difficult to know if a given activity is functional or not, we will use promiscuity in the most general sense (Copley 2017; Peracchi 2018; Nath and Atkins 2008). The more promiscuous an enzyme is, the more reactions it catalyses, regardless of whether these activities are functional or not.

Enzyme promiscuity has multiple possible mechanical and chemical causes (Nobeli et al. 2009; Khersonsky and Tawfik 2010). A prominent one is that proteins can fluctuate between different, energetically equivalent conformations (Campbell et al. 2016; Nobeli et al. 2009; James and Tawfik 2003; Khersonsky and Tawfik 2010). These alternative conformations alter the shape of an enzyme’s active site and thus which substrates fit into this site (Ben-David et al. 2012). Evolution can change enzyme activities by stabilising some conformations and destabilising others (Campbell et al. 2016). For example, over billions of years, $\beta$-lactamases evolved from flexible promiscuous enzymes into the current more rigid enzymes that are efficient and specific catalysts of penicillin breakdown. This loss of flexibility came at the cost of losing activity for other antibiotics (Zou et al. 2015). Conversely, directed evolution of a metallo-$\beta$-lactamase towards the antibiotic cephalexin can result in a more flexible and promiscuous enzyme (Tomatis et al. 2008).

Promiscuity can also occur within the same conformation, for example, because an alternative substrate may be able to bind to the active site, albeit imperfectly (Nobeli et al. 2009). Certain substrates are so similar that enzymes cannot discriminate between them (Peracchi 2018) and require additional proofreading mechanisms outside the active site to do so. Examples include some aminoacyl-tRNA synthetases that have to discriminate between very similar amino acids when attaching them to their cognate tRNA (Tawfik and Gruic-Sovulj 2020).

Promiscuity has two important consequences for enzyme evolution. First, it facilitates the evolution of new metabolic pathways when organisms encounter a novel environment. The required catalytic activities do not need to evolve from scratch, but can be recruited from the side reactions catalysed by existing enzymes (Glasner et al. 2020; D’Ari and Casadesús 1998; Newton et al. 2018; Conant and Wolfe 2008; Peracchi 2018). Second, promiscuity can affect the fate of gene duplicates, affecting, for example, the survival of duplicates or the acquisition of novel functions (Conant and Wolfe 2008; Noda-Garcia and Tawfik 2020; Des Marais and Rausher 2008; Sikosek et al. 2012). Duplication with subsequent changes in catalytic activity of either duplicate is common during enzyme evolution (Copley 2020).

Whilst most duplicates quickly become lost (Lynch and Conery 2000), the fate of surviving duplicates is shaped by their enzymatic activities and the selection pressures acting upon them. Some duplicates benefit an organism by simply increasing the expression of a low-efficiency enzyme (Bergthorsson et al. 2007; Kondrashov and Kondrashov 2006). In others where the duplicated enzyme catalyses two beneficial reactions that strongly trade-off with one another, duplication can allow each duplicate to subfunctionalise, that is, to retain a subset of the functions of the generalist ancestor and to specialise by improving the catalysis of one of the two competing reactions (Noda-Garcia and Tawfik 2020; Des Marais and Rausher 2008; Sikosek et al. 2012). Subfunctionalization can also occur without such a trade-off and without increasing catalytic activity. In such cases, the duplicates of a bi-functional ancestor can experience a release from selection for one of the two activities, which subsequently become eroded by loss-of-function mutations and genetic drift (Force et al. 1999). Subfunctionalisation is also at times followed by the gain of a new function in one of the duplicates, a process known as neofunctionalisation (Conant and Wolfe 2008; Scannell and Wolfe 2008). It can be facilitated by promiscuity (Glasner et al. 2020), which can buffer the effects of deleterious mutations that decrease functional activities, allowing duplicates to accumulate more mutations, with each mutation increasing the chance of discovering new promiscuous activities (Glasner et al. 2020).

A poorly understood factor in the survival and evolution of duplicate enzymes is the effect of mutations on an enzyme’s promiscuity itself. There is strong evidence that mutations constrain catalytic activities for different reactions, i.e. their effects on different catalytic activities are correlated (Bayer et al. 2017; Tawfik and Gruic-Sovulj 2020; Savir et al. 2010; Kaltenbach and Tokuriki 2014). Such constraints usually also entail trade-offs, i.e. a high catalytic rate for one reaction implies a low rate for the other reactions (Khersonsky and Tawfik 2010; Tawfik and Gruic-Sovulj 2020; Tokuriki et al. 2012; Kaltenbach et al. 2016). Trade-offs can be strong but are more often weak (Kaltenbach and Tokuriki 2014; Aharoni et al. 2005; Gould and Tawfik 2005; Tokuriki et al. 2012; Des Marais and Rausher 2008; McLoughlin and Copley 2008).

The strength of a trade-off can influence the fate of a duplicated gene. If trade-offs between two important enzyme activities are sufficiently weak, selection may not favour subfunctionalisation strongly enough to prevent one duplicate from becoming lost (Noda-Garcia and Tawfik 2020).

As opposed to trade-offs, some constraints on enzyme activities create synergies between two or more reactions (Espinosa-Cantú et al. 2015; Savir et al. 2010; van Loo et al. 2019). For example, in some enzymes an increase in the catalytic activity of the enzyme for one substrate requires that it also improves its activity on another substrate (van Loo et al. 2019). In other enzymes the catalytic rates of different reactions are even inseparable such that the catalysis of one reaction entails the catalysis of another reaction, even if that other reaction is deleterious (Savir et al. 2010). Such constraints are less well documented but may also affect enzyme evolution after duplication.

Constraints may interact with selection pressures on enzyme activity to affect enzyme evolution. Here, we explore the possibility that this interaction can render an enzyme a poor catalyst even when selection favours high catalytic activity. This can occur when multiple activities of an enzyme are in irresolvable conflict with one another. In this case, the enzyme can be considered to be in a state of *frustration* (Ferreiro et al. 2014). Frustration is a concept originally describing the suboptimal arrangements of atoms in glasses, which cannot achieve the optimal regular arrangement that define crystals due to conflicting forces affecting their orientation (Ferreiro et al. 2014). Frustration occurs at multiple levels of biological organisation (Ferreiro et al. 2014; Wolf et al. 2018). It has, to our knowledge, not been studied for promiscuous enzymes.

We follow a three-pronged approach to explore how constraints may have shaped the evolution of enzyme promiscuity. First, we examine the distribution of promiscuity amongst enzymes reposited in the Braunschweig Enzyme Database (BRENDA) (Jeske et al. 2019). To do so, we quantify the degree of substrate promiscuity in terms of how well a given enzyme catalyses reactions with different substrates (Nath and Atkins 2008). If these catalytic efficiencies for different substrates are similar, we consider the enzyme a generalist. If they are dissimilar, with the enzyme acting much more efficiently on one substrate than on others, we consider it a specialist. We find that the distribution of promiscuity is bimodal, with enzymes being largely either specialists or generalists. Second, we use a simple biophysical model to show that enzyme biochemistry alone cannot explain the bimodality we observe in empirical enzymes. Third, we build a phenomenological model of how constraints affecting the ability of an enzyme to catalyse multiple reactions influences the degree of specialisation that is possible before and after duplication. This model is based on experimental case studies of constraints in ribozymes, enzymes, and other proteins engaged in two activities (Bendixsen et al. 2019; Kaltenbach and Tokuriki 2014; Tokuriki et al. 2012; Lite et al. 2020; van Loo et al. 2019; Savir et al. 2010). Our results suggest that the bimodal distribution of enzyme promiscuity observed in empirical data cannot be explained solely by enzyme biochemistry but also involves selection followed by duplication.

## Results

### Promiscuous Enzymes Have a Bimodal Distribution of Promiscuity

**Fig. 1:**
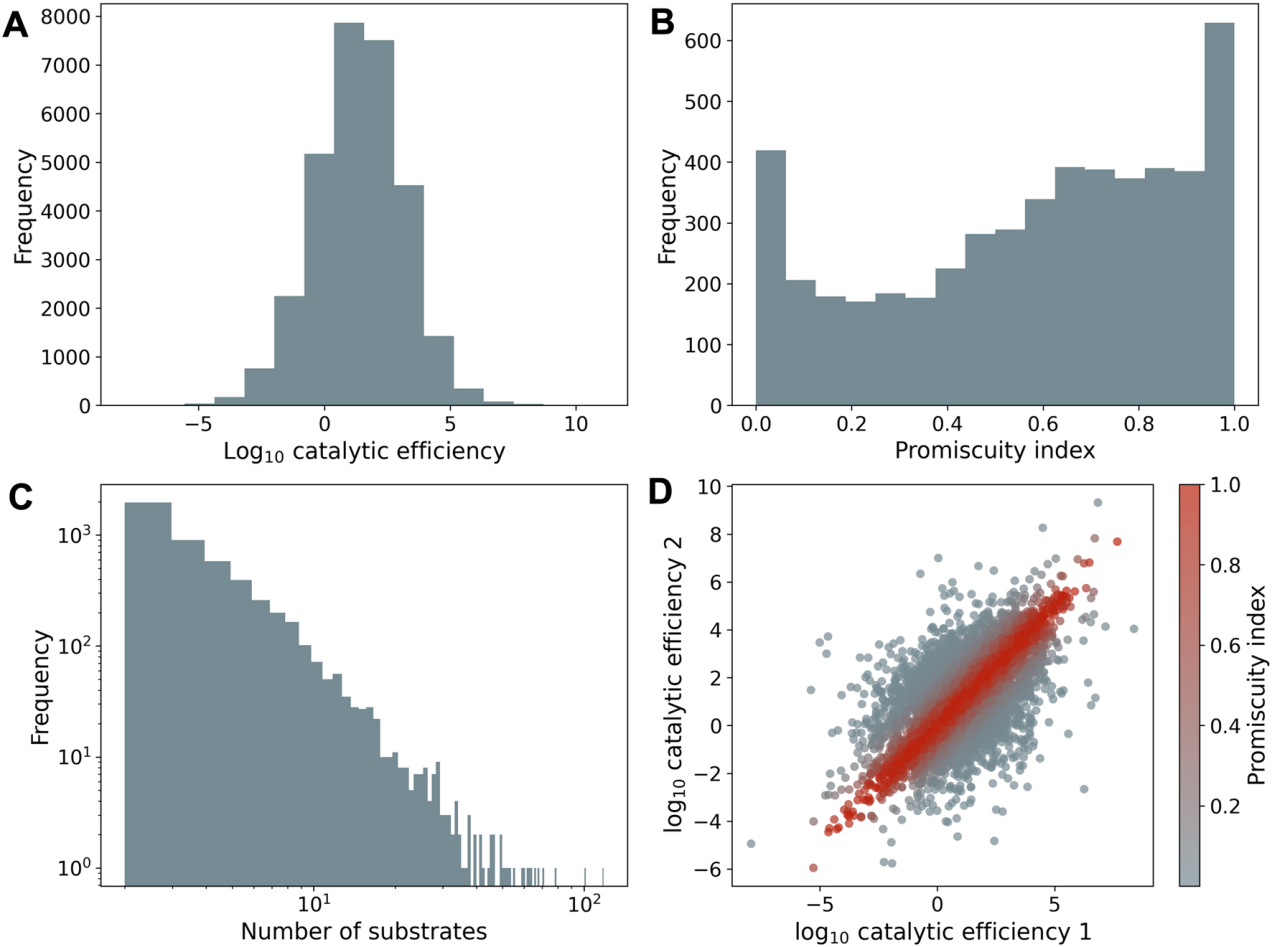
The distribution of promiscuity indices for enzymes in BRENDA are bimodal, and the logarithm of catalytic efficiencies of reactions catalysed by the same enzyme are correlated. **A** Distribution of 30,184 catalytic efficiencies in the BRENDA dataset (Jeske et al. 2019). **B** Distribution of the promiscuity index (Nath and Atkins 2008) (methods equation 1) of 5028 enzymes for which catalytic parameters (Michaelis constant and turnover number) are available for at least two substrates. We estimated the promiscuity index of each enzyme from the catalytic efficiency (turnover divided by Michaelis constant) for each substrate of a reaction that the enzyme catalyses. **C** Distribution of the number of substrates (natural and non-natural) per enzyme used to estimate the promiscuity index for each of the 5028 enzymes. **D** The logarithm of catalytic efficiencies of two substrates whose reactions are catalysed by the same enzyme are correlated. For this analysis, we sampled at random without replacement two substrates for those enzymes with more than two substrates in the database (see methods). Each circle corresponds to one of the 5028 enzymes; colour indicates the promiscuity index (high in red and low in grey) of the enzyme for the two sampled substrates

We started by considering the distribution of promiscuity amongst enzymes in nature. To do so, we obtained catalytic parameters from the BRENDA enzyme database (Jeske et al. 2019), and compiled a dataset of 30,184 substrates with measurements for both the turnover number and Michaelis constant. From these measurements we calculated catalytic efficiencies (turnover divided by Michaelis constant) as a measure of how well an enzyme catalyses a given reaction (Eisenthal et al. 2007). The median turnover number in our dataset is 5.49 s^-1^, the median Michaelis constant is $1.99\times 10^{-1}$ mM, and the median catalytic efficiency is 26.0 mM^-1^ s^-1^, which is lower than in a previous report (Bar-Even et al. 2011) because we also analysed non-natural substrates. Consistent with this report (Bar-Even et al. 2011), we found that the distribution of catalytic efficiencies is log-normal (Fig. 1A).

After pre-processing and quality control steps (methods), our dataset contained data for 5028 enzymes with catalytic parameters for at least two substrates. These enzymes come from 1621 species from all three domains of life and from viruses. 2039 of these enzymes have a protein accession number that allows their identification in protein databases (methods). For each protein in this dataset, we calculated a promiscuity index (Nath and Atkins 2008) from the catalytic efficiencies of its reactions with each substrate. This index quantifies how similar or dissimilar an enzyme’s catalytic efficiencies are for multiple substrates. It ranges between the limits of one (reactions with all substrates are catalysed with very similar efficiencies) and zero (reactions with all but one substrate are catalysed with zero efficiency). We found that the distribution of promiscuity index values is bimodal (Fig. 1B). This bimodal distribution remains when we considered data for only the 2039 enzymes with a protein accession number (Online Resource 1 figure S1). 39 percent of all promiscuity index values are based on two substrates per enzyme (Fig. 1B). The average enzyme has 4.90 known substrates ($\pm 6.00$ standard deviation) with measured catalytic parameters. We considered the possibility that the promiscuity of an enzyme reflects how much effort has gone into characterising its substrate promiscuity. However, this is not the case, because the number of substrates reported per enzyme is not associated with the promiscuity index (Kendall’s $\tau =6.01\times 10^{-3}$, $p=0.557$, $n=5028$).

We found that highly promiscuous enzymes tended to be slightly less efficient catalysts. Specifically, highly promiscuous enzymes have lower catalytic efficiencies for the reactions they catalysed best than less promiscuous enzymes (Pearson’s $r=-0.21$, $p=7.57 \times 10^{-52}$, $n=5028$). In addition, we found that the logarithmically (base 10) transformed catalytic efficiencies of reactions with different substrates catalysed by the same enzyme were moderately similar (Pearson’s $r=0.617$, $p=0.00$, $n=5028$; for enzymes with more than two substrates, two substrates were chosen at random without replacement, see methods for details on this analysis, Fig. 1D).

We also searched for orthologs in our dataset and found that younger orthologs tend to have slightly more similar promiscuities. For the 2039 enzymes with a protein accession number, we searched for possible orthologs by downloading amino acid sequences from Uniprot (The UniProt Consortium 2023) and running a BLAST search (Drost et al. 2015; Camacho et al. 2009) (Online Resource 1 section S1.10). We found 6,683 pairs of putative orthologs. For 6,602 of these enzyme pairs, we correlated the percent sequence identity from the BLAST search with the absolute difference in promiscuity index of the enzymes (Kendall’s $\tau = -0.0318, z=-3.87, p=1.07 \times 10^{-4}, n=6602$). We found that enzymes with similar sequences have weakly more similar promiscuities.

Based on these observations we formulated three hypotheses that may explain why the empirical distribution of promiscuity indices is bimodal. Our first hypothesis is that the distribution is the consequence of ascertainment or measurement bias. We modelled a scenario in which the discovery of new substrates of an enzyme is biased towards substrates with similar catalytic efficiency to those already known (Online Resource 1 section S1.7). In this case, as more substrates are discovered, the bimodality of the promiscuity distribution disappears. Given that our dataset contains catalytic parameters of about five substrates per enzyme (on average), this first hypothesis is not well supported (Online Resource 1 section S1.7). The second hypothesis, which we explore in the next section, is that enzyme biochemistry may suffice to explain the bimodality. The third, alternative hypothesis posits that evolutionary and biochemical factors may explain the bimodality of the promiscuity distribution. We explore these factors in the subsequent sections.

### A Biophysics-Based Null Distribution of Enzyme Promiscuity

In this section, we aim to establish a null distribution for the promiscuity index. We will employ this null distribution to evaluate whether enzyme promiscuity can exhibit a bimodal distribution solely due to inherent variation in enzymatic efficiencies or if additional factors are necessary to explain this bimodality. We sought to establish this distribution for a protein capable of catalysing two reactions. Using Michaelis–Menten kinetics, we were able to derive a formula that estimates the enzyme catalytic efficiencies from the activation free energy of enzymatic reactions, $\Delta G_{1}^{\#}$ and $\Delta G_{2}^{\#}$ (see Online Resource 1 section S1.6 for details). We then use the promiscuity index equation (methods, equation 1) to calculate the promiscuity from these catalytic efficiency estimates.

Consequently, the distribution of enzyme promiscuity is conditional on the distribution of the activation free energy of enzymes, which is empirically known. It approximates a Gaussian shape with a mean spanning from $-4$ to $-7$ kcal/mol and a standard deviation of approximately 2 kcal/mol (Sousa et al. 2020). We then used a sampling process and sampled $10^3$ pairs of $\Delta G_{1}^{\#}$ and $\Delta G_{2}^{\#}$ from the known empirical distribution of activation free energies, and for each pair calculated the enzyme promiscuity. We then examined the distribution of enzyme promiscuity using this process by fitting a beta distribution to these distributions. We selected the beta distribution for modelling these distributions because it is commonly used to represent the probability distribution of variables when the distribution type is unknown. This distribution involves two positive shape parameters, alpha and beta, whose combination determines the shape and skewness of the distribution.

**Fig. 2:**
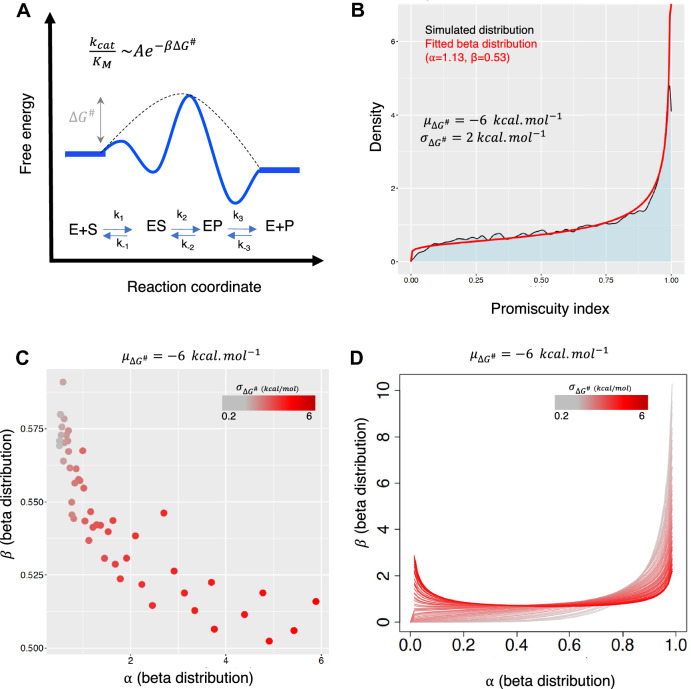
Enzyme promiscuity distribution inferred from enzyme kinetics. **A** The Michaelis–Menten kinetics and the relationship between enzyme promiscuity and the activation free energy of the enzymatic reaction. **B** The distribution of enzyme promiscuity for natural enzymes with the average activation free energy $\mu _{\Delta G^{\#}}=-6$ kcal/mol and $\sigma _{\Delta G^{\#}}=2$ kcal/mol. The black and red curves correspond to the simulated distribution and the fitted distribution with a beta distribution, respectively. **C** The shape parameters of fitted beta distributions for enzyme promiscuity values obtained from distributions of activation free energies with different standard deviation changing from 0.2 kcal/mol (grey) to 6 kcal/mol (red). **D** Corresponding beta distributions to the distributions presented in panel C

Figure 2A demonstrates that for different values of $\Delta G_{1}^{\#}$ and $\Delta G_{2}^{\#}$ sampled from the activation free energy distribution of natural enzymes, the promiscuity index distribution is unimodal. This suggests that the variation in enzyme kinetics alone is insufficient to account for the presence of distinct categories of enzymes—generalist and specialist—leading to a bimodal enzyme promiscuity distribution. Additional factors are likely necessary to generate such enzyme diversity. To model the impact of these factors, we augmented the variation in activation free energy. We assumed that mechanisms contributing to a bimodal distribution achieve this by amplifying the variance in enzymatic free energies. Indeed, we observed that the distribution of enzyme promiscuity index adopts a bimodal shape when the variation in activation free energy is substantially heightened, at least doubling from $\sigma _{\Delta G^{\#}}$ kcal/mol to $\sigma _{\Delta G^{\#}} > 4$ kcal/mol (Fig. 2B, C).

In summary, our basic biophysical model demonstrates that a bimodal enzyme promiscuity distribution is more likely to arise due to evolutionary factors, rather than being solely a consequence of enzyme kinetics creating a distribution where both generalist and specialist enzymes coexist.

### A Selection-with-Constraints Model of Enzyme Evolution

**Fig. 3:**
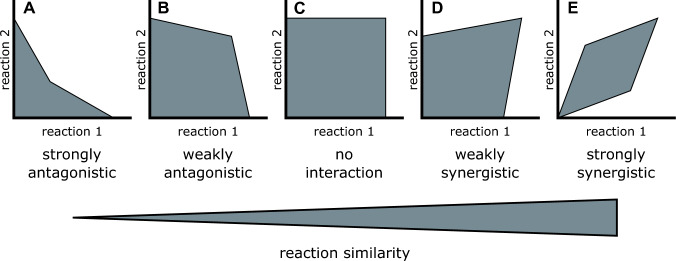
Schematic of our constraint-based model of the range of possible relationships between two reactions catalysed by the same enzyme. These possible relationships range from antagonism to synergism, and are here subdivided into five possible classes: **A** strong antagonism, **B** weak antagonism, **C** no interaction/unconstrained, **D** weak synergism, and **E** strong synergism. Each axis denotes the catalytic efficiency of the enzyme for one of the two reactions. The grey areas contain the feasible combinations of catalytic efficiencies, which are delimited by constraints (thin black lines). These feasible spaces circumscribe the possible catalytic efficiencies of different sequence variants of the same enzyme. All combinations of catalytic efficiencies within a feasible space are reachable by mutation, whereas catalytic efficiencies outside the feasible space are not. The pairwise relationships can be arrayed in order of increasing similarity of the reactions, although reaction similarity is not necessarily the only factor influencing the shape of the pairwise relationships

We next explore evolutionary factors that may explain why the promiscuity distribution is bimodal. Specifically, we explore three factors that are known to influence the evolution of enzymes: Selection on enzyme activities, constraints on these activities, and gene duplication. Our evolutionary hypothesis is that selection favours enzymes with higher catalytic efficiencies, but catalytic efficiencies are subject to constraints. Gene duplication can simplify the selective pressures acting on an enzyme, allowing the duplicates to escape or bypass some of the constraints limiting the evolution of the pre-duplication enzyme. The result of these three factors acting together is a bimodality in promiscuity. To explore this hypothesis, we developed a simple phenomenological model, based on empirical case studies (see next paragraph), of how multiple activities of the same enzyme can interfere with one another and constrain the evolution of the enzyme. To start with, we identified a range of possible pairwise relationships between the catalytic ability of an enzyme for two reactions (Fig. 3). For simplicity, we have assumed that the pairwise relationships are symmetrical (i.e. both reactions are constrained in the same way) and created a set of linear constraints approximating these pairwise relationships. These constraints limit which combinations of catalytic efficiencies are possible for a given enzyme, which produce a continuous space we termed the *feasible space* of efficiencies. Our model makes no assumptions about the causes of these constraints. However, based on empirical studies discussed in the next paragraphs, we interpret these constraints as the result of biophysical or biochemical limitations on catalysis. As such, we interpret the feasible space outlined by these constraints to delimit all possible genotypes. It contains all possible sequence variants of an enzyme that catalyse at least one of the two reactions in question. In other words, it contains all catalytic efficiencies for the two reactions that are reachable by mutation.

Our constraints range from strongly antagonistic, where a high catalytic efficiency for one reaction entails low efficiency for the other, to strongly synergistic, where high efficiency for one reaction can only be achieved when there is also high efficiency for the other. These pairwise relationships are based on empirical case studies of both proteins and ribozymes. For example, strong antagonism (Fig. 3A) exists amongst RNA molecules that are either self-cleaving or ligases, but cannot attain high efficiency for both activities at once (Bendixsen et al. 2019). Weaker trade-offs (Fig. 3B) are commonly observed in experiments where an enzyme is subjected to multiple rounds of selection for a promiscuous function (Kaltenbach and Tokuriki 2014), for example, from a phospotriesterase to an arylesterase (Tokuriki et al. 2012). In a study (Lite et al. 2020) investigating protein-protein binding between the anti-toxin ParD3 and two toxins that are closely related to each other, ParE3 and ParE2, mutations in the anti-toxin allow it to bind either one or both toxins. In this system, all combinations of specificities are possible without much of a trade-off (Fig. 3C). For some members of the alkaline phosphatase enzyme family (van Loo et al. 2019), at high catalytic efficiency, the catalytic efficiency of one reaction can only be increased if a mutation also simultaneously increases the efficiency of the other reaction. In contrast, at lower catalytic efficiency mutations can increase or decrease the efficiencies independently of one another. This example motivates the weak synergism shown schematically in Fig. 3D. Finally, enzymes like Rubisco catalyse harmful side reactions whose efficiency can only be reduced by mutations that also reduce the efficiency of the main reaction (Fig. 3E; (Savir et al. 2010)). These five pairwise relationships (Fig. 3) are the fundamental and discrete units of our model.

Where a pair of catalytic efficiencies lies on the spectrum from antagonistic to synergistic constraints may depend on the similarity of the underlying reactions. However, this interpretation requires caution, because the ability to catalyse multiple reactions is a complex trait influenced by many different properties of an enzyme’s active site and of the reactants, and there may thus be no straightforward measure of ‘reaction similarity’ (Babtie et al. 2010; Janzen et al. 2020).

Selection with constraints is not sufficient to explain the bimodal distribution of promiscuity amongst empirical enzymes. As stated above, we assume in our model that selection favours increasing the catalytic activity of the enzyme with respect to the beneficial reactions. Many enzymes exhibit diminishing returns epistasis with regards to catalytic efficiency, i.e. a decrease in enzyme activity causes a larger fitness loss than the fitness gain resulting from an increase in activity (Yi and Dean 2019; Chou et al. 2014). We include this diminishing returns epistasis in our model (Online Resource 1 section S1.4). Consequently, promiscuous enzymes catalysing multiple beneficial reactions will evolve into generalists with high promiscuity. For example, in enzymes catalysing two reactions that are strongly antagonistic with respect to one another, even if higher catalytic efficiency could be achieved in one reaction by decreasing catalytic efficiency for the other reaction, such specialisation would cause a net decrease in fitness (see next section). Thus, the evolution of specialist each catalysing one of these beneficial reactions requires a third factor next to selection and constraints: Gene duplication.

Duplication is a major mode of enzyme evolution and can affect the fate of promiscuous enzymes, as discussed earlier in this work. Enzyme evolution is characterised by high rates of gene duplication followed by subfunctionalization of the duplicates (Sikosek et al. 2012; Noda-Garcia and Tawfik 2020). Consequently, we modelled a scenario in which the single copy gene encoding a generalist ancestral enzyme that catalyses multiple reactions undergoes multiple rounds of duplication. We assumed that each duplicate is under positive selection to catalyse only one of the reactions catalysed by the generalist. In other words, each duplicate is subject to selection for a different reaction and its abilities to catalyse the other reactions do not affect fitness. We assumed that catalytic activities not under selection will tend to disappear due to loss-of-function mutations fixed by genetic drift (Force et al. 1999), resulting in subfunctionalization even in the absence of selection. This inactivation can occur relatively quickly, within $10^6$ generations (Force et al. 1999). An enzyme’s ability to catalyse a given reaction will only remain if its loss is prevented by an interplay of selection and the constraints we model (Fig. 3). Consequently, we report the minimum catalytic efficiencies for each non-functional activity permitted by constraints and selection on functional catalytic activities. We assumed that natural selection favours increasing the catalysis of every functional reaction. We studied whether duplicated enzymes are always more efficient catalysts than their generalist ancestor. We also asked to what extent duplication can reduce or remove obstacles to evolving more efficient enzymes. In addition, we investigated the degree of promiscuity in duplicated enzyme variants.

In the next two sections, we will examine some of the evolutionary consequences of this selection-constraints-duplication model and then consider to what extent it may account for the bimodal distribution of promiscuity in empirical enzymes.

### Catalytic Constraints can Create ‘Frustrated’ and Promiscuous Enzymes

**Fig. 4:**
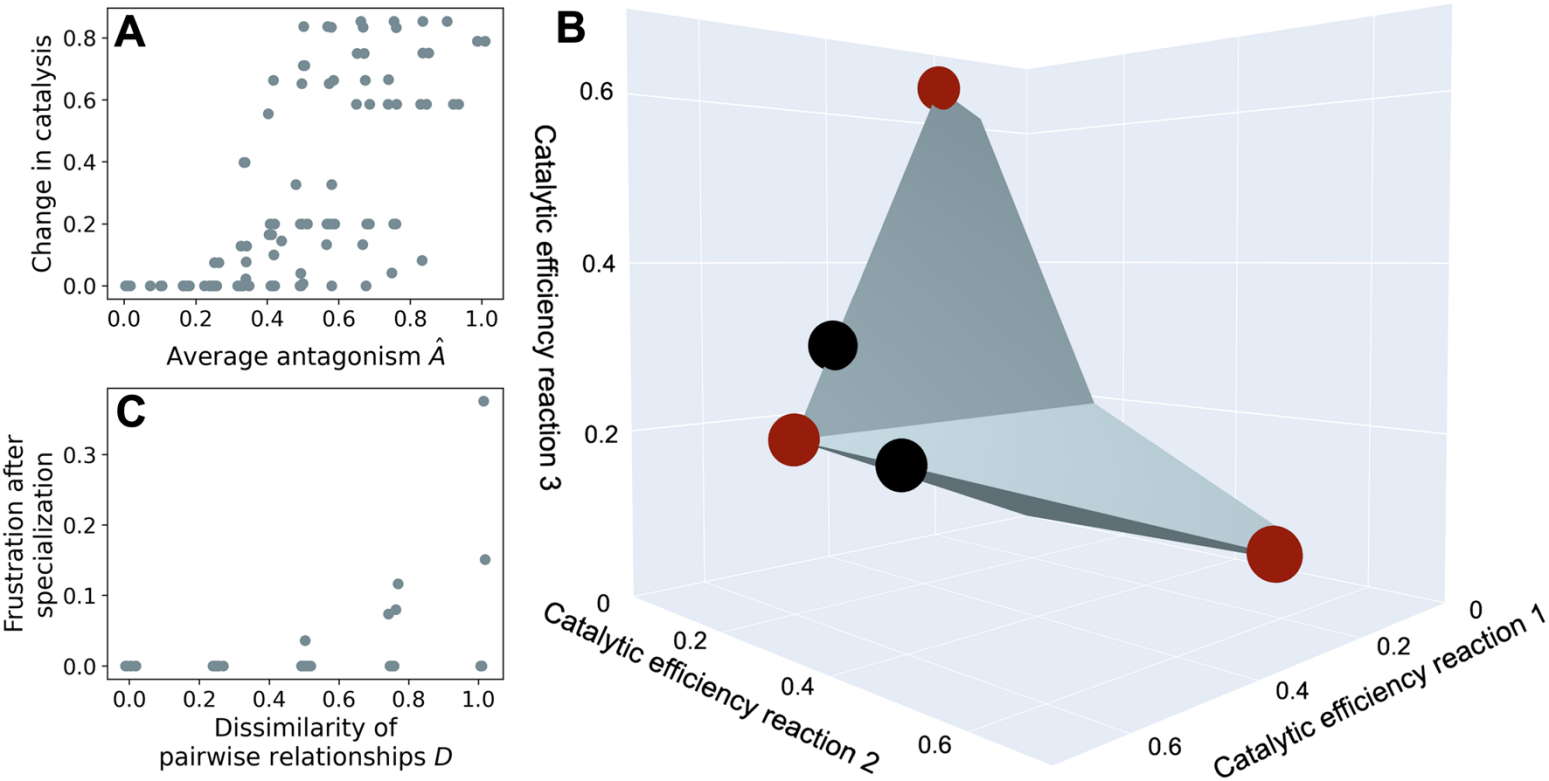
Catalytic constraints can create ‘frustrated’ and promiscuous enzyme variants. **A** As feasible spaces of enzymes contain increasingly less synergistic and more antagonistic interactions, the gain in catalytic activity of enzyme variants due to duplication and specialisation increases. Feasible spaces are constructed from combinations of the pairwise relationships in Fig. 3 (methods). Catalytic activity is scaled between zero (no activity) to one (maximum activity). We quantified the extent to which different feasible spaces are characterised by strongly synergistic or strongly antagonistic relationships between their reaction pairs by quantifying how antagonistic the relationships are on average (methods). To quantify this average antagonism score ${\hat{A}}$, we scored each of our pairwise relationships (Fig. 3) with an antagonism score *A* ranging from one (strongly antagonistic, Fig. 3A) to zero (strongly synergistic, Fig. 3E) in increments of 0.25. The average antagonism score ${\hat{A}}$ that characterises a feasible space is the average of the antagonism scores *A* of each of the pairwise relationships that constitute the feasible space. Consequently, ${\hat{A}}=0$ denotes that all three pairwise relationships are strongly synergistic (Fig. 3E), whilst ${\hat{A}}=1$ denotes that all pairwise relationships are strongly antagonistic (Fig. 3A). **B** Hypothetical example of the feasible space of a promiscuous enzyme with high frustration both before and after frustration. This enzyme has a feasible space (grey polygon) that does not permit the evolution of highly efficient catalysis for any of the reactions it catalyses, i.e. the theoretical maximum catalytic efficiency of one is outside the feasible space. The reason is that the enzyme catalyses two reactions that are strongly antagonistic to one another but are strongly synergistic to a third reaction. The structure of this feasible space does not allow the elimination of catalytic activity for any one reaction unless the enzyme is entirely inactivated. Black and red circles show the two enzyme variants maximising fitness before duplication and after duplication, respectively. **C** As the pairwise relationships that constitute a feasible space become less similar, duplication and specialisation becomes less able to resolve frustration. We quantify the extent of frustration as the difference between the catalytic activity of an enzyme variant that maximises fitness within the bounds of the feasible space and the catalytic activity that characterises an optimal enzyme, which equals one. If all reactions catalysed by an enzyme are subject to selection, we quantify the frustration of the enzyme as the mean frustration of all its reactions. We quantify the dissimilarity *D* of pairwise relationships constituting the feasible space of an enzyme using the antagonism scores *A* of the pairwise relationships (methods). A dissimilarity of $D=0$ means that all three pairwise relationships are the same (for example, all are strongly synergistic), whilst $D=1$ means that the pairwise relationships are highly dissimilar (for example, the combination plotted in **B**). All results in **A** and **C** are shown for the 105 possible specialised enzymes predicted from feasible spaces with three reactions

We investigated how selection, constraints and duplication may drive the evolution of promiscuous enzymes by comparing the ancestors and the duplicates predicted by our constraint-based model. We first considered the case of an enzyme that can catalyse three reactions, because this is the lowest number of reactions where the pairwise constraints can interfere with one another. To simulate enzymes that can catalyse more than $n=2$ reactions, we combined the pairwise relationships in Fig. 3 to form higher-dimensional feasible spaces that contain all possible combinations of catalytic efficiencies of the enzyme’s *n* reactions that can be reached by mutation. We investigated all feasible spaces that could be constructed using these five pairwise relationships. For enzymes with $$\left( {\begin{array}{c}n\\ 2\end{array}}\right) =k$$ reaction pairs, we assigned from this set of five relationships a set of constraints for each of the *k* reaction pairs of the enzyme (methods). For an enzyme catalysing three reactions, there are $k=3$ reaction pairs. Because the same pairwise relationship can occur multiple times (sampling with replacement) and the ordering is not important, the five possible pairwise relationships (Fig. 3) combine to form $C(5+3-1,3)=35$ feasible spaces (see also Online Resource 1 section S1.5). These 35 feasible spaces comprise every possible unique combination of the five pairwise relationships between the enzyme’s three reactions (methods). For example, in one feasible space all three reactions may be strongly synergistic with respect to one another and in another strongly antagonistic. In a third feasible space, one pair of reactions (*a* and *b*) may be strongly antagonistic, the second pair (*b* and *c*) unconstrained, and the third pair (*a* and *c*) weakly antagonistic. These feasible spaces contain every possible enzyme variant catalysing reactions at efficiencies permitted by the constraints set out by the pairwise relationships.

We then searched for those enzyme variants in every feasible space that maximised fitness (methods). We did so in two steps. First, we identified in every feasible space those enzyme variants that could act as generalist ancestors. These enzyme variants are under selection to catalyse all three reactions as efficiently as possible given the constraints of the feasible space. Depending on the shape of the feasible space, multiple enzyme variants may fulfil this requirement. Consequently, there are more possible ancestors (41) than feasible spaces (35). The reason that there are more ancestor enzyme variants than feasible spaces is that feasible spaces that contain strong antagonism between at least two reactions contain more than one ancestor enzyme variant with equivalent fitness, but different catalytic efficiencies for the three reactions. In our model, the fitness of an ancestor depends on all three reactions and is more sensitive to low catalytic efficiencies because we assumed a fitness function with diminishing returns (methods). Consequently, selection will push ancestors to preferentially catalyse one of the two (or three) reactions trading-off with one another, but without losing catalysis for the other reaction(s). Because all reactions are equally important for achieving high fitness, which reaction is preferred is arbitrary and therefore, multiple enzyme variants can have equivalent fitness.

Second, we identified in every feasible space those enzyme variants that could act as duplicates. Every duplicate is a descendant of the ancestor enzyme, a descendant that has come under selection to catalyse only one reaction. Given that there are 35 feasible spaces and three possible duplicates per feasible space, there are $3 \times 35 = 105$ possible duplicates.

We identified the constraints that permit duplicates to evolve into more efficient catalysts than their ancestors. We observed that, on average, as pairwise relationships become more antagonistic, ancestor enzyme variants become poorer catalysts (Pearson’s $r=-0.718$, $p=1.25\times 10^{-7}$, $n=41$). Consequently, as the pairwise relationships become more antagonistic, duplication and specialisation results in increasingly large improvements in catalysis (Fig. 4A, Pearson’s $r=0.680$, $p=1.42\times 10^{-15}$, $n=105$). The reason is that for enzymes whose constituent reactions are all weakly or strongly synergistic, being a generalist already results in optimal catalysts. Duplication and specialisation entail no further improvement. Conversely, because strong antagonism permits high activity for one substrate only if another reaction is poorly catalysed, it causes adaptive conflict between different selection pressures. The result is that a generalist ancestor enzyme variant where all three activities are functional and strongly antagonistic to one another will be a suboptimal catalyst for all three reactions. By analogy to similar phenomena in physics and protein biochemistry we say that such an ancestral enzyme is in a state of frustration (Wolf et al. 2018; Ferreiro et al. 2014). We quantified the frustration of an enzyme variant before and after duplication as the difference between the realised catalytic efficiency and the maximum possible catalytic efficiency averaged across all reactions the enzyme is selected for. This measure of frustration is independent of the total number of reactions catalysed by the enzyme. Frustration in the form of adaptive conflict can in principle be resolved through duplication and subsequent specialisation of the duplicates. Our results indicate that this is indeed the case, with enzymes that have strong antagonism between their abilities to catalyse reactions benefiting the most from duplication (Fig. 4A).

However, duplication cannot always eliminate conflict between different activities of the same enzyme variant. Even when an enzyme variant is not subject to adaptive conflict because selection is acting on only one reaction, constraints from other reactions can still interfere with the catalysis of that reaction. For example (Fig. 4B), frustration cannot be entirely resolved in an enzyme that catalyses three reactions, of which two reactions strongly trade-off with each other but both are strongly synergistic with the third reaction. This feasible space does not permit much specialisation, because specialisation is prohibited by the strong synergism, nor is it possible within the feasible space to reach high catalytic efficiency, which would require specialisation, a requirement set by the strong antagonism. Thus any enzyme variant in this space will be frustrated and promiscuous. An interesting property of the enzyme in this example, and of enzymes that remain frustrated after duplication in general, is that it catalyses reactions whose pairwise relationships violate an expectation set by ‘reaction similarity’. Previously, we discussed the possibility that the relationship between two reactions is due to the similarity of their reaction mechanisms, with strongly synergistic pairs of reactions being very similar, and strongly antagonistic pairs of reactions being very dissimilar. For the example we just discussed (Fig. 4B), if reaction *a* is strongly antagonistic with regards to (i.e. very different from) reaction *b*, and reaction *b* is strongly synergistic with regards (i.e. very similar) to reaction *c*, then we may expect that reaction *c* is very different from reaction *a* and that their relationship is strongly antagonistic. Strong synergism between reactions *a* and *c* violates this expectation. Weaker violations of reaction similarity also result in constrained enzymes that remain frustrated after duplication and specialisation. Enzymes with less similar pairwise relations between reactions (Fig. 4C) are more likely to remain frustrated even after duplication. Overall, 71 percent of our 35 feasible spaces contained frustrated enzyme variants before duplication, and 17.

An important consequence of frustrated enzyme variants with a strongly constrained feasible space is that these enzyme variants are promiscuous and poor catalysts even for the reaction in which they specialise (e.g. Fig. 4B). Indeed, enzyme variants that are more promiscuous tend to have, on average, slightly lower catalytic efficiency for the reaction they catalyse best (Pearson’s $r=-0.264$, $p=6.52\times 10^{-3}$, $n=105$). This association becomes stronger as the number of reactions per enzyme increases (Online Resource 1 table S2). We note that the association is weak, because some enzyme variants that are highly promiscuous catalyse multiple reactions at high efficiency. For example, if all reactions are synergistic with respect to one another, high efficiency catalysis for one reaction entails high efficiency catalysis for the other two reactions.

Overall, these observations suggest three classes of enzymes. The first comprises enzymes that are not frustrated and highly promiscuous, where duplication is not necessary for the evolution of high efficiency because all reactions are synergistic (or unconstrained) with respect to one another. The second comprises enzymes that are frustrated, but where frustration can be resolved through duplication, for example, if all reactions are strongly antagonistic to one another. The third comprises frustrated enzymes where frustration is not entirely resolvable due to interfering constraints (as in the example of Fig. 4B).

As the number of reactions per enzyme increases, interference between constraints becomes increasingly probable and the proportion of enzymes with irresolvable frustration increases dramatically (Online Resource 1 figure S2). This occurs because the number of pairwise relationships increases quadratically with each additional reaction. Specifically, the number of pairwise relationships increases with the number of reactions *n* in accordance with $O(n^2)$. Consequently, the probability that an enzyme’s ability to catalyse a reaction has pairwise relationships with other reactions of the enzyme that violate the expectation of reaction similarity increases dramatically and therefore the probability that the enzyme experiences frustration.

### Frustration Caused by Catalytic Constraints Produces Bimodal Distributions of Enzyme Promiscuity

**Fig. 5:**
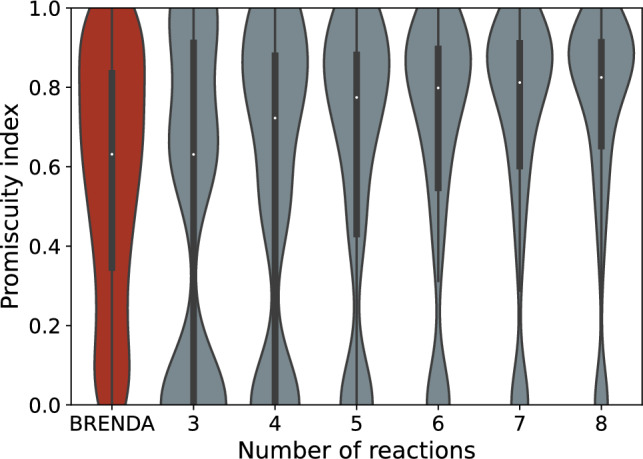
The constraint-based model predicts a bimodal distribution of promiscuities even as the number of reactions per enzyme increases. White dots show the median promiscuity index, boxes contain the 25 and 75 percentiles, and vertical lines contain all promiscuity index values within 1.5 times the interquartile range. The violin plots are Gaussian kernel density estimates of the distributions of promiscuity index values. The distribution of promiscuity indices of empirical enzymes from BRENDA (Jeske et al. 2019) is shown in red, the promiscuity indices of specialised enzyme variants predicted by our model for enzymes catalysing between three and eight reactions in grey. The number of enzymes in the BRENDA dataset is 5028. Multiple enzyme variants exist for each feasible space. For the model predictions, the number of enzyme variants is (from three to eight reactions per enzyme): 105, 840, 5005, 23256, 88550, and 287680

In the previous section, we observed that our constraint-based model predicts the evolution of both specialists and generalists. It should therefore be able to account for the bimodal distribution of promiscuity of empirical enzymes. We investigated if it could by considering the distribution of promiscuities predicted by our model after duplication, because the generalist state is often frustrated and natural selection will act to preserve duplicates and favour their specialisation. Consequently, we assumed that the generalist state is generally transient. The promiscuity distribution of model enzymes with fewer reactions is more similar to that of the empirical enzymes (Fig. 5), although the distributions are not the same (e.g. three reactions, two-sample Kolmogorov–Smirnov test, $D_{105, 5028}=0.4$, $p=2.36\times 10^{-15}$). As the number of reactions catalysed by an enzyme increases in our model, the distribution of promiscuities increasingly skews towards higher promiscuities.

Importantly, however, the predicted promiscuity distribution is bimodal at both low and high number of reactions per enzyme. In other words, enzymes preferentially have low or high promiscuity. At the low end of this bimodal distribution are highly specialised enzymes. These are enzymes whose reactions are sufficiently unconstrained that their promiscuity decays after duplication through mutation and drift or frustrated enzymes whose frustration can be resolved by duplication. At the high end are frustrated enzymes whose frustration cannot be resolved or promiscuous generalists in which specialisation yields no benefit. In sum, these results suggest that constraints limiting the ability of enzymes to catalyse multiple reactions, and the frustration these constraints cause, may be an important factor in the evolution of promiscuous enzymes.

## Discussion

We investigated the extent of enzyme promiscuity by studying catalytic parameters of 5028 enzymes from the BRENDA database (Jeske et al. 2019). Enzymes in this data set catalysed reactions with an average of 5 substrates. This number is almost certainly an underestimate. Experimental studies that sampled enzymatic substrates have shown that enzymes can catalyse reactions with a much larger number of substrates (Copley 2017; Khersonsky and Tawfik 2010). For example, a survey assessed the ability of 217 members of the haloalkanoic acid dehalogenase superfamily to catalyse reactions with 167 substrates (Huang et al. 2015). Whereas, only 24 percent of the enzymes were relatively specialised and catalysed reactions with fewer than 6 substrates, 47 percent were intermediate generalists (6-40 substrates), and 23 percent were strong generalists (41-143 substrates). We employed a promiscuity index that quantifies to what degree an enzyme catalyses its reactions at equal or unequal rates (Nath and Atkins 2008). The distribution of this index amongst our 5028 enzymes is bimodal, with enzymes either being specialists (one reaction catalysed at a substantial higher rate) or generalists (multiple reactions catalysed at similar rates), with fewer intermediates.

We investigated several hypotheses that may explain this bimodality. We first considered and ruled out ascertainment bias, and we determined that enzyme biochemistry does not suffice to explain the observed biochemistry. We next turned to an evolutionary explanation involving natural selection, constraints, and gene duplication. Specifically, we hypothesised that mutations may constrain the evolution of promiscuous enzymes, so that enzymes catalysing several reactions can only evolve some combinations of catalytic activities but not other combinations. We represented these constraints in a qualitative model inspired by empirical case studies of constraints in promiscuous proteins and ribozymes engaged in two activities (Bendixsen et al. 2019; Kaltenbach and Tokuriki 2014; Tokuriki et al. 2012; Lite et al. 2020; van Loo et al. 2019; Savir et al. 2010).

Our model, which considers how enzymes evolve, predicts a bimodal promiscuity distribution. Specifically, our model predicts that the evolution of a bimodal promiscuity distribution requires that natural selection drives the evolution of increasing catalytic efficiency in enzymes, that gene duplication permits some enzymes to specialise, and that constraints on enzyme promiscuity exist.

Our model also makes several empirically verifiable predictions about how the relationships between different activities catalysed by the same enzyme may affect the evolution of the enzyme by duplication and subfunctionalization. One of them is that some enzymes catalyse sets of reactions that are simultaneously subject to strong trade-offs between them, without the ability to lose catalysis for any one of them. In other words, these enzymes catalyse reactions that are simultaneously incompatible and mutually irremovable. For such enzymes, our model predicts that mutation does not permit specialisation of the enzyme. Additionally, these hypothetical enzymes would not be able to catalyse any of the reactions with high catalytic efficiency. Given that this state is the result of mutually incompatible constraints and selection pressures, we drew an analogy to the physics of spin glasses (Ferreiro et al. 2014), where opposing forces prohibit the arrangement of atoms in the regular arrangement of a crystal. Instead, the arrangement of atoms is ‘frustrated,’ and the atoms form an irregular glass consisting of many energetically equivalent arrangements, none of which is optimal. Examples of frustration can be found throughout biological systems (Wolf et al. 2018), for example, in the energetics of protein folding (Ferreiro et al. 2014). Our model suggests that frustration amongst promiscuous enzymes falls into two kinds. The first is frustration that can be resolved by duplication. The second cannot be resolved by duplication (Fig. 4).

Unfortunately, we are not aware of any examples of frustrated enzymes in the literature. We believe that this absence is a consequence of how little is in fact known about the relationships between multiple catalytic activities of the same enzyme, and how mutation changes these relationships. However, there is some circumstantial evidence that frustration may play a role in the evolution of enzyme promiscuity in the way our model suggests, and our model also suggests ways to test for frustration. One line of evidence comes from incomplete subfunctionalisation (Lynch and Force 2000), which is common amongst moonlighting enzymes (Espinosa-Cantú et al. 2015) and amongst duplicated paralogs involved in metabolism in *Saccharomyces cerevisiae* (DeLuna et al. 2008). Whilst not all these cases will be the result of frustration, it should be possible to identify frustrated proteins through deep mutational scanning (Araya and Fowler 2011). In such proteins, no mutation would be able to eliminate overlap in activities without inactivating the protein entirely. Another line of evidence is that our model predicts that frustrated, promiscuous enzymes tend to be slightly poorer catalysts. Indeed, we found this association between promiscuity and activity amongst enzymes in the database.

Our model implies that frustration is both a creative and a limiting force in the evolution of promiscuous enzymes, just as it is on other levels of biological organisation (Wolf et al. 2018). When frustration can be resolved through duplication it can be a driver of the evolution of specialised and efficient enzymes. Conversely, irresolvable frustration can limit the evolution of efficient catalysts. Our results are similar to findings in multifunctional gene regulatory networks, where the gain of one function by a regulatory network can make it more difficult for the network to gain another function (Payne and Wagner 2013).

Our results also imply that the absolute number of activities catalysed per enzyme may be important for enzyme evolution. The same reaction might be catalysed by different enzymes that have different constraints and different extents of frustration. This is plausible because some reactions are catalysed by enzymes with very different active sites and evolutionary histories (Davidi et al. 2018). We speculate that selection is likely to favour those enzymes that catalyse fewer reactions, namely for two reasons. First, our results show that enzymes that catalyse fewer reactions are less likely to be frustrated, thus permitting the evolution of specialisation and high catalytic efficiency. Second, whenever side reactions catalysed by an enzyme are deleterious, enzymes that are frustrated may be unable to lose these deleterious reactions and remain functional. Over time, these effects may lead to an enrichment of enzymes that catalyse few reactions and are less promiscuous. This evolutionary force may be counteracted by the greater likelihood that enzymes with many promiscuous reactions are recruited into nascent metabolic pathways (Glasner et al. 2020; D’Ari and Casadesús 1998; Newton et al. 2018; Conant and Wolfe 2008; Peracchi 2018). Which of these two factors is more important for enzyme evolution is an interesting avenue of future research.

Like other models, ours contains several simplifying assumptions. First, because we aimed to explore how constraints affect enzyme promiscuity, we ignored other factors that can limit the power of selection to increase beneficial catalytic activities. In other words, we modelled selection as a process of optimization. However, selection has rarely produced ‘perfect’ enzymes, i.e. enzymes limited only by substrate diffusion (Khersonsky and Tawfik 2010; Davidi et al. 2018; Bar-Even et al. 2011). Most enzymes catalyse reactions with efficiencies orders of magnitude below the diffusion limit (Bar-Even et al. 2011), even though many enzymes could become more efficient catalysts with only few mutations (Davidi et al. 2018). Theoretical studies of selection acting on metabolic enzymes show that selection is strongly limited by diminishing returns epistasis (Newton et al. 2015, 2018; Labourel and Rajon 2021; Kacser and Burns 1981). We note that our assumption of selection as optimization does not affect our qualitative results, because constraints between different activities can in principle also affect enzymes with only moderate activity.

Second, our model assumes additive interactions between constraints, but constraints may often interact non-additively. Catalysis depends on the precise position of amino acids at an enzyme’s active site and throughout the rest of the protein (Wrenbeck et al. 2017) and on the motion of the enzyme during catalysis (James and Tawfik 2003; Campbell et al. 2016; Zou et al. 2015). Consequently, the multi-dimensional constrained spaces of possible mutations we modelled are at best rough approximations of real-life feasible spaces. In addition, the model assumes that every kind of constraint, and combination of constraints, is equally probable. In reality, some constraints may be more common than others, changing the probability of encountering frustration in enzyme catalysis. For example, if enzymes are biased towards catalysing similar reactions, then we may expect frustrated enzymes to be less common than predicted by our model. Unfortunately, we know very little about the constraints acting on multiple reactions at once. Few studies consider the effect of mutation on two activities at once (Bendixsen et al. 2019; Kaltenbach and Tokuriki 2014; Tokuriki et al. 2012; Lite et al. 2020; van Loo et al. 2019; Savir et al. 2010) and even fewer consider three or more activities (Wrenbeck et al. 2017; Bayer et al. 2017; Zhang et al. 2012; Markin et al. 2021). Given this lack of knowledge we preferred to keep our model simple. Future empirical work on enzymatic constraints may reveal fascinating deviations from our naive expectations.

Third, we did not include neofunctionalisation (Conant and Wolfe 2008; Scannell and Wolfe 2008) in our model. Neofunctionalisation could easily be integrated as a process that adds new reactions to an enzyme after a duplication event. However, evidence suggests subfunctionalization plays a substantially larger role in enzyme evolution than neofunctionalisation (Glasner et al. 2020), with enzymes already being promiscuous or multifunctional before duplication. We therefore decided to keep this study simple and only investigate subfunctionalization.

Fourth, we only modelled catalytic efficiencies of one enzyme, but other aspects of enzyme evolution may be at least as important to explain promiscuity (Copley 2021). For example, many of the initial mutations increasing the activity of a promiscuous enzyme occur in other genes. They include mutations affecting the concentrations of substrates or inhibitors of the promiscuous enzyme (Kim et al. 2019; Morgenthaler et al. 2019). Some such mutations may affect the activity of an enzyme competing for the same substrate (Kim et al. 2019) or increase the expression of an upstream enzyme producing the substrate in question (Morgenthaler et al. 2019). Such mutations may be necessary for low-efficiency catalytic reactions to become subject to selection (Copley 2021). They can also decrease the selection pressure required for the evolution of more efficient, specialised enzymes. In addition, changing environments may favour the emergence and maintenance of promiscuity. The uncertainty of natural environments may be one of the reasons why free-living organisms have more promiscuous enzymes than intracellular organisms (Martínez-Núñez and Pérez-Rueda 2016).

Finally, we have limited our model to catalytic activities that are beneficial, because we were interested in the extent to which constraints limit subfunctionalisation. However, many promiscuous reactions may be deleterious. For example, metabolic enzymes catalyse side reactions whose products are often useless or actively harmful to a cell, and need to be removed (Peracchi 2018). Selection against deleterious reactions may be an important driver of specialisation (Noda-Garcia and Tawfik 2020), as is the case for aminoacyl-tRNA synthetases (Tawfik and Gruic-Sovulj 2020).

All these limitations render our model simple, and this simplicity has an advantage. It means that the model can be easily generalised to other levels of biological organisation, where a given component is involved in more than one activity, and where selection favours specialisation (Rueffler et al. 2012). Consider organismal development, which involves developmental modules that can undergo duplication and can be subject to selective pressure to engage in multiple activities. Examples include teeth, which have become differentiated to serve various roles in mammals (Weiss 1990) and arthropod legs, which became specialised for both locomotion and feeding over time (Boxshall 2004). Constraints and frustration may play an important role in the evolution of biological systems on multiple levels of organisation.

## Methods

### BRENDA Data Curation

We downloaded enzyme kinetic data from BRENDA (Jeske et al. 2019) (https://www.brenda-enzymes.org, Accessed 3 April 2020) and wrote custom scripts to extract the substrates (where available) of each enzyme, as well as the turnover number and Michaelis constant for each of the substrates. Whenever multiple estimates for the turnover number and Michaelis constant were available for the same substrate, we used the average of all estimates for that particular substrate. Because substrate names are not standardised in BRENDA, multiple synonyms of the same substrate may be used for the same reaction. To alleviate this problem, we identified synonyms for individual substrates using data from the Kyoto Encyclopedia of Genes and Genomes (KEGG) database (Kanehisa and Goto 2000) and replaced these in our dataset with a unique substrate name. As in previous studies (Bar-Even et al. 2011), we removed common cofactors from our substrate list, because in most cases these molecules are not the primary substrates of an enzyme. In addition, they may be affected by different evolutionary pressures (Bar-Even et al. 2011). Specifically, we removed entries for the five most common cofactors (ATP, NAD+, NADPH, NADH, and NAP+) from our dataset. Unlike earlier work (Bar-Even et al. 2011), we kept both natural and non-natural substrates, because we were interested in studying the potential for both reaction and substrate promiscuity. In this way, we obtained 30,184 entries from BRENDA. Each entry contains the turnover number and Michaelis constant associated with a given substrate.

BRENDA is organised according to enzyme commission numbers (EC), which classify enzymes by the kind of reaction they catalyse (Jeske et al. 2019). Many enzymes also have protein accession numbers, which allow identification of the enzyme in other databases, such as GenBank (Benson et al. 2013).

If a protein accession number was available, we used all catalytic efficiencies of the enzyme for further analysis, including for different reactions. In rare cases (6 out of 3899 protein accessions) a given protein accession number appeared in more than one species. We attributed this to a labelling error and discarded the data associated with these enzymes.

Unfortunately, 65 percent of the entries in the available data did not have a protein accession number and therefore can only be identified in terms of the reaction catalysed (the EC number of the reaction) and the species expressing the enzyme. For such enzymes, we assumed that a given EC number for a species in BRENDA represented a single enzyme, although we cannot exclude that a single BRENDA entry may refer to multiple enzymes or that reactions catalysed by the same enzyme are represented under different EC numbers, unless a protein accession number was supplied.

For all enzymes where the Michaelis constant and the turnover number is reported for at least two substrates, we calculated the catalytic efficiency for each substrate (turnover divided by Michaelis constant, mM^-1^ s^-1^). Overall, our dataset comprises 5028 enzymes that fulfil this criterion, of which 2039 have a protein accession number. From these catalytic efficiencies, we calculated the promiscuity index below (Nath and Atkins 2008) from the catalytic efficiencies of these 5028 enzymes.

### Promiscuity Index

We calculated the promiscuity index (Nath and Atkins 2008) as

1 $$\begin{aligned} I = -\frac{1}{\log n} \sum _{i=1}^{n} \frac{x_i}{\sum _{j=1}^{n} x_j} \log \frac{x_i}{\sum _{j=1}^{n} x_j} \end{aligned},$$

where *n* is the number of reactions with different substrates per enzyme and *x* is the catalytic efficiency of the enzyme with respect to a given reaction. The promiscuity index quantifies the similarity or dissimilarity between the catalytic efficiencies of an enzyme for the reactions it catalyses. To do so, it draws on the concept of entropy as a way to quantify the ‘diversity’ of catalytic efficiencies analogous to the way in which entropy is used as a measure of species diversity in ecosystems (Nath and Atkins 2008). The maximum entropy is $\log n$, which corresponds to an enzyme that catalyses all reactions with equal efficiency. To scale the promiscuity index between zero (only one of the reactions is catalysed by the enzyme) and one (all reactions are catalysed equally well), we divide the entropy by a factor of $1 / \log n$. We used this index to estimate the promiscuity of empirical enzymes but also of simulated enzymes generated through random sampling (Online Resource 1 section 1.7) and of enzymes predicted by our constraint-based model.

### Constraint-Based Model Description

Our model takes a constraint-based approach to the relationships between two or more reactions catalysed by the same enzyme. We described different variants of the same enzyme catalysing *n* reactions as points in an *n*-dimensional space of catalytic efficiencies. We divided this catalytic efficiency space into a feasible and an infeasible space. The feasible space contains all combinations of catalytic efficiencies that can be reached by mutation (i.e. all possible variants of the enzyme in question). The infeasible space contains those catalytic efficiencies that cannot be reached. A set of constraints defines the limits of the feasible space. In our model, the most basic form of these constraints describe the relationship between a pair of reactions. This relationship can range from strongly antagonistic—high catalytic efficiency for one reaction entails low efficiency for the other—to strongly synergistic—high efficiency for one reaction entails high efficiency for the other. Between these two extremes lies a spectrum of intermediates, with enzymes that can catalyse both reactions at high efficiency or only one of them. For simplicity, we kept pairwise relationships symmetrical, so that the constraints on one reaction are the same as on the other, although asymmetrical relationships may exist (e.g. (Bendixsen et al. 2019)). In addition, we scaled catalytic efficiencies to lie between zero (no activity) to one (maximum possible catalytic efficiency) for any one reaction. For some high-performing enzymes, this limit will correspond to the diffusion limit, corresponding to a catalytic efficiency of approximately $10^{9}$ s^-1^ M^-1^ (Bar-Even et al. 2011). Details on these pairwise constraints and how they interact to form higher-dimensional feasible spaces are given in Online Resource 1 section S1.5. The constraints themselves are listed in Online Resource 1 table S1.

We used this model to simulate two scenarios. In the first scenario, all *n* reactions are catalysed by the same enzyme. Every reaction is beneficial, and we assumed that natural selection acts to increase the catalytic efficiency of the enzyme for all *n* reactions. In the second scenario, the enzyme has undergone multiple rounds of duplication and subfunctionalization, so that there are *n* duplicates. Each duplicate is under selection for a different reaction such that one reaction is subject to selection per duplicate (details in Online Resource 1 section S1.4). The catalytic efficiencies of a duplicate for the other reactions are neutral (for a case where selection does still act on the ability of the duplicates to catalyse more than one reaction, see Online Resource 1 section S1.8). We compare the catalytic efficiencies of these enzyme variants before and after the duplication events.

Because we assumed that natural selection increases catalytic efficiencies, we modelled the action of selection as an optimization problem that maximises fitness by increasing catalytic ability (for details see Online Resource 1 section S1.5). Given that for many real-life traits fitness is more sensitive to decreasing than to increasing activity, we chose a fitness function with diminishing returns (Online Resource 1 section S1.4). We identified the enzyme variants maximising fitness within a given feasible space through non-linear programming (Online Resource 1 section S1.5). In addition, some duplicated enzyme variants catalyse reactions that are not under selection. For these neutral reactions, we identified the range of catalytic efficiencies that could evolve without affecting the catalysis for the reaction that is still under selection. We did so by performing a variability analysis (Online Resource 1 section S1.5). For enzymes that have undergone duplication and specialisation, we used the minimum of the catalytic efficiencies predicted by the variability analysis for the reactions not under selection to determine to what extent loss-of-function mutations can erode these neutral activities.

To quantify the frustration in the ability of an enzyme variant preferred by natural selection to catalyse a reaction, we computed the difference between the variant’s maximum catalytic efficiency for the reaction under selection (predicted by optimization in the *n*-dimensional feasible space) and the maximum possible catalytic efficiency of one. For an enzyme variant catalysing multiple reactions before duplication, we reported the average of these frustration values as an indicator of the frustration of the enzyme variant with respect to all its reactions. For duplicated enzymes, we quantified the frustration of each specialised duplicate with regards to the reaction it was selected for. By comparing the average frustration before and after duplication, we could infer to what extent duplication can help resolve frustration.

We scored feasible spaces according to where they are positioned on a spectrum between pure strong synergy, where all pairwise relationships between reactions are strongly synergistic, to pure strong antagonism, where all relationships are strongly antagonistic. We assigned each of our five pairwise relationships (Fig. 3) an antagonism score *A* going from strong synergism (zero) to strong antagonism (one) in increments of 0.25, so that pairwise relationships that are unconstrained have an antagonism score of 0.5. For each feasible space, we calculated the average antagonism score ${\hat{A}}$ of all pairwise relationships that constitute the feasible space as

2 $$\begin{aligned} {\hat{A}} = \frac{1}{k} \sum _{i=1}^{k} A_i \end{aligned},$$

where *k* is the total number of pairwise relationships and $A_i$ is the antagonism score of reaction pair *i*. The average antagonism score ${\hat{A}}$ lies in the range between one (pure strong antagonism) to zero (pure strong synergism).

We also compared how dissimilar the antagonism scores of the pairwise relationships that constitute a feasible space are. We defined a dissimilarity score *D* of a feasible space, which we computed from the antagonism scores of the pairwise relationships *A* that constitute the feasible space so that

3 $$\begin{aligned} D = \sum _{i=1}^{k-1} \sum _{j=i+1}^{k} \mid A_i - A_j \mid \end{aligned},$$

where *k* is the total number of pairwise relationships, and $A_i$ is the antagonism score of reaction pair *i*. We reported the dissimilarity score of a given feasible space *D* relative to the dissimilarity score $D_{\text {max}}$ of the feasible space with the highest dissimilarity score and the same number of reactions per enzyme *n*. This rescaling allowed us to report the dissimilarity *D* on a scale of zero (all pairwise relationships are the same) to one (all pairwise relationships are as different as possible).

### Supplementary Information

Below is the link to the electronic supplementary material.

Supplementary file 1 (PDF 734 KB)

## Data Availability

Code for the main optimization model, analysis, and figure plotting is available at https://github.com/michaelacmschmutzer/enzymepromiscuity.
