# Supplementary material for: Frustration can Limit the Adaptation of Promiscuous Enzymes Through Gene Duplication and Specialisation
Source: J Mol Evol. 2024 Mar 12;92(2):104–20. doi: 10.1007/s00239-024-10161-4 (PMC10978624; doi:10.1007/s00239-024-10161-4)
Supplement: Supplementary file 1 — (PDF 734 KB) [file 239_2024_10161_MOESM1_ESM.pdf]

Supplementary materials to "Frustration can limit the adaptation of promiscuous enzymes through gene duplication and specialisation", Journal of Molecular Evolution

Michael Schmutzer, Pouria Dasmeh, Andreas Wagner*

February 15, 2024

* Department of Evolutionary Biology and Environmental Studies, University of Zurich, Zurich, Switzerland. Swiss Institute of Bioinformatics, Lausanne, Switzerland. Santa Fe Institute, Santa Fe, New Mexico, USA. andreas.wagner@ieu.uzh.ch

S1 Supplementary materials

S1.1 Supplementary tables

Relationship	Constraints
Strong synergism	$-3x_1 + 1x_2 + 0 \leq 0$
	$-1x_1 + 2x_2 - 1 \leq 0$
	$1x_1 - 3x_2 + 0 \leq 0$
	$2x_1 - 1x_2 - 1 \leq 0$
Weak synergism	$-1x_1 + 5x_2 - 4 \leq 0$
	$5x_1 - 1x_2 - 4 \leq 0$
Unconstrained	$0x_1 + 1x_2 - 1 \leq 0$
	$1x_1 + 0x_2 - 1 \leq 0$
Weak antagonism	$1x_1 + 4x_2 - 4 \leq 0$
	$4x_1 + 1x_2 - 4 \leq 0$
Strong antagonism	$1x_1 + 4x_2 - 1 \leq 0$
	$4x_1 + 1x_2 - 1 \leq 0$

Table S1: Linear constraints delimiting the pairwise relationships between catalytic efficiencies x_1 and x_2 for two different reactions catalysed by the same enzyme.

Number of reactions	Pearson's r	p-value	n
3	-0.264	6.52×10^{-3}	105
4	-0.365	8.15×10^{-28}	840
5	-0.407	8.69×10^{-199}	5005
6	-0.426	0.0	23256
7	-0.436	0.0	88550
8	-0.443	0.0	287680

Table S2: The promiscuity index of enzyme variants after duplication and specialisation is negatively correlated with the activity of the reaction best catalysed by that variant. This association increases as the number of reactions potentially catalysed per enzyme increases. Reported are the strength of this association (Pearson's r with p-value) and the number of specialised enzyme variants n. All values are based on predictions of the constraint-based model.

S1.2 Supplementary figures

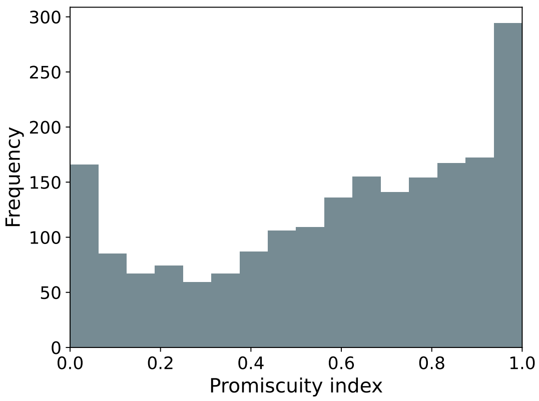

Figure S1: The distribution of promiscuity indices [1] for enzymes with a protein accession in BRENDA is bimodal. The distribution is shown for 2039 enzymes with reported catalytic parameters for at least two substrates and a protein accession.

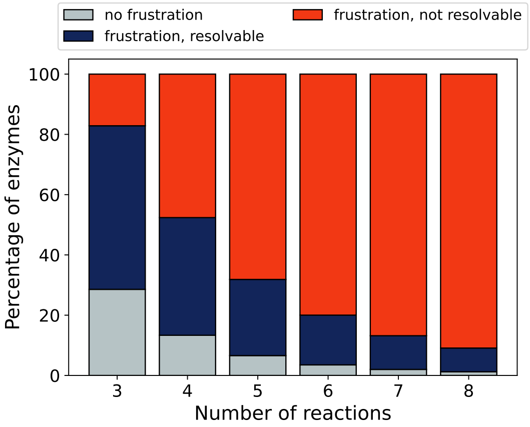

Figure S2: Irresolvable frustration becomes increasingly common as enzymes catalyse more reactions. Stacked bar plot of the percentage of enzymes whose feasible spaces contain enzyme variants that are not frustrated before or after duplication (grey), enzyme variants that are frustrated before duplication, but where duplication resolves frustration and different enzyme variants can specialise for different reactions (blue), and enzyme variants where duplication cannot entirely resolve frustration (orange). Data is based on 35, 210, 1001, 3876, 12650, and 35960 possible model enzymes with between 3 and 8 reactions, respectively.

S1.3 Generating samples from multivariate log-normal distributions

To investigate how the amount of available empirical data might influence the promiscuity index, we modelled the distribution of catalytic efficiencies as a multivariate log-normal distribution. To parametrise this distribution, we needed to estimate the covariation between the catalytic efficiencies of reactions with different substrates catalysed by the same enzyme. For the purpose of estimating this covariance, we sub-sampled our filtered BRENDA dataset so that each enzyme has data on only two substrates, so that we could correlate the catalytic efficiencies of these substrates. If an enzyme had only two substrates we used these substrates for our analysis. If an enzyme had more than two substrates, we chose two of them at random without replacement. We then took the logarithm (base 10) of the catalytic efficiencies in the sub-sampled dataset, because the resulting distribution is known to be approximately normal [2]. We computed the mean and standard deviation of these logarithmically (log) transformed catalytic efficiencies. For the bivariate distribution, we calculated the covariance matrix directly from the sub-sampled, log-transformed dataset. For the multivariate distributions, we assumed that the covariances between the logarithm of catalytic efficiencies are the same between all reactions and extrapolated from the bivariate data. Consequently, for a multivariate distribution with n variates, we estimated the correlation coefficient from the covariance between the two sub-sampled, log-transformed catalytic efficiencies. We assumed that the correlations between all of the n variates is the same, and thus placed this correlation coefficient into all non-diagonal entries of a correlation matrix A of size $n \times n$, and set the diagonal entries to one. We then multiplied each entry in the correlation matrix A by the product of the two standard deviations of the two sets of empirical log-transformed catalytic efficiencies in the dataset to obtain the covariance matrix.

We used the resulting mean, standard deviation, and covariate matrices to parametrise the bivariate and multivariate log-normal distributions (up to eight dimensions) and drew 5000 samples at random from every distribution to simulate how the promiscuity index depends on the amount of data available. For comparison, we also sampled in the same way from uncorrelated multivariate log-normal distributions with the same mean and standard deviation. We then calculated the promiscuity index [1] for both the correlated and the uncorrelated samples.

S1.4 Fitness function

Our fitness function is a modification of Kacser & Burns [3] model of flux F through a linear pathway consisting of n enzymes, each catalysing one reaction

$$F = \frac{C}{1/E_1 + 1/E_2 + \cdots + 1/E_i + \cdots + 1/E_n}, \tag{1}$$

where C is a constant proportional to the difference in concentration of the reactants and products, and each E stands for the catalytic activity of one enzyme. E can be approximated as the product of the enzyme's abundance and its catalytic efficiency x [4, 5]. We assumed that the fitness f is equal to the flux F. We set C equal to the number of reactions n, and all enzyme abundances to one, so that the maximum attainable fitness f when all catalytic steps are catalysed optimally is equal to one. In summary, our fitness function can be interpreted as depending on the flux through a linear pathway, where each step in the pathway is catalysed by the same promiscuous

enzyme or a set of specialised enzymes. We note that this fitness function is not linear in the enzyme efficiencies.

We simulated the effect of subfunctionalization after duplication by simplifying our fitness function for each duplicate. Before duplication, there is one enzyme catalysing all n reactions, and we modelled the action of selection on that enzyme as a single optimisation problem with a 'generalist' fitness function, equivalent to equation 1, that contains a total of n variables, where each variable stands for the activity of the enzyme E_i for the reaction i. After duplication, there are n duplicate enzymes, and because each duplicate is under selection for only one reaction, there are now n optimisation problems. The 'specialist' fitness function of each duplicate contains only a single variable E_i for the one reaction i it is being selected for, i.e. equation 1 with a single term ($n = 1$). This subdivision of the fitness function allowed us to simulate the gene-specific selection pressures driving subfunctionalization.

S1.5 Optimization in high-dimensional feasible spaces

We identified enzyme variants that maximize fitness in the feasible space by solving the following nonlinear programming problem, which has m inequality constraints:

$$\begin{aligned} &\text{maximize } f(x) \\ &\text{subject to } g_i(x) \leq 0 \text{ for } i \in (1,\ldots,m) \\ &\qquad\qquad x \in X \\ &\qquad\qquad x \geq 0. \end{aligned} \tag{2}$$

Here, X is a vector (x_1, x_2,..., x_N) of catalytic efficiencies of each of N reactions catalysed by an enzyme, and where $f(x)$ is the fitness function in equation 1, or one of its simplified, specialist versions. The inequality constraints $g_i(x)$ are linear combinations of the elements of the vector X. The m inequality constraints correspond to the lines delimiting the feasible spaces given in table S1 and outlined in figure 3 in the main text.

We specified all our inequality constraints for two reactions at a time, and defined five different sets of these constraints that describe different possible relationships between pairs of enzyme activities (figure 3, table S1). To construct higher-dimensional feasible spaces, we used combinations of our five pairwise constraint relationships, and we modelled all possible combinations of these relationships. Because different reaction pairs that constitute a feasible space can have the same pairwise relationship, and because a feasible space can contain more pairwise relationships $k = \binom{n}{2}$ between the n catalysed reactions than the five pairwise relationships we have defined (figure 3), the total number of feasible spaces corresponds to $C(5,n) = \binom{5+k-1}{k}$. For example, for an enzyme catalysing three reactions, we investigated $C(5,3) = 35$ different feasible spaces. We require that every feasible space with $n > 2$ reactions conforms to *all* pairwise constraints.

As an example, assume that an enzyme catalysing $n = 3$ reactions a, b, and c, which are all unconstrained with respect to one another (figure 3c), i.e. the pairwise constraints are all in the shape of a square. The feasible space of this enzyme will be a cube that permits enzyme variants that catalyse the three reactions with combinations of catalytic efficiencies such as (1,1,1), (1,1,0) or (0,1,0). Another example is an enzyme with almost the same set of constraints as the previous one, except that the relationship between reactions a and b is strongly antagonistic

(figure 3a). For this second enzyme, the combinations of catalytic efficiencies (1,1,1) and (1,1,0) lie outside the feasible space. However, combinations such as (1,0,1) and (0,1,1) are still possible. In these two examples, every pairwise combination of catalytic efficiencies permitted by one of the pairwise constraints can be found somewhere in the three-dimensional feasible space. However, there are some feasible spaces where different pairwise relationships will 'cut' into each other, i.e. combinations of catalytic efficiencies permitted by one relationship will be prohibited by another. In such cases, feasible spaces obey the more restrictive constraint. For example, an enzyme catalysing three reactions may have a strongly synergistic (figure 3e) relationship between reactions a and b, whereas the other relationships may be unconstrained as in the previous two examples. Strong synergism between two reactions entails that it is not possible to eliminate catalytic activity for one reaction except by also eliminating activity for the other reaction. Consequently, the strong synergism between reactions a and b means that the point (0,1,1) lies outside the feasible space, despite the fact that the unconstrained relationship between b and c would permits it.

Nonlinear programming requires a convex space, i.e. a space in which a straight line can connect any two points within the feasible space without leaving the feasible space. However, the strongest form of trade-off we consider (strong antagonism) creates a concave space that violates this convexity requirement. To avoid this issue we used a 'divide and conquer' approach. When a given feasible space contains one or more strongly antagonistic trade-offs, we divide the resulting nonconvex space into smaller and partially overlapping convex subspaces (figure S3). In n=2 dimensions, subdividing the concave feasible space into two convex spaces yields two subspaces. For $n > 2$ dimensions (reactions), the number of subspaces can range between two to n. The maximum number of subspaces is n and corresponds to the case where every pairwise relationship is strongly antagonistic. In this case, every dimension will have its own subspace.

We solved the nonlinear optimisation problem for each subspace. In the case of a single enzyme catalysing n reactions, we solved a single optimisation in each subspace using the generalist fitness function. For duplicated enzymes, we solved for every specialist fitness function in every subspace, i.e. if all relationships are strongly antagonistic, we solved $n \times n$ optimisation problems. In either case, every optimisation returns a solution within a given subspace. We ranked these different solutions and retained only those that have the highest fitness (figure S3). Different solutions from different subspaces may have the same fitness, and in those cases we accepted more than one solution.

The solutions found through nonlinear programming maximize fitness within the constraints of the feasible space. However, for duplicated enzymes, the catalytic efficiencies for reactions that are not selected can vary, without this variability changing the catalytic efficiency of the one reaction that is subject to selection for a given duplicate. This may occur when a solution lies not on a vertex of the boundaries of the feasible space, but on an edge or a plane. To estimate the extent of the variability in the catalytic efficiencies of non-selected reactions, we performed a variability analysis similar to flux variability analysis in metabolic modelling [6]. Briefly, this method consists of adding an equality constraint ($h(x) = 0$) to the optimization problem that specifies that the fitness function must remain at the maximum (i.e. $h(x) = f(x) - f_{sol}$, where f_{sol} is the maximum fitness determined previously). We then performed additional optimizations that maximise and minimise the catalytic efficiency for each reaction subject to this additional

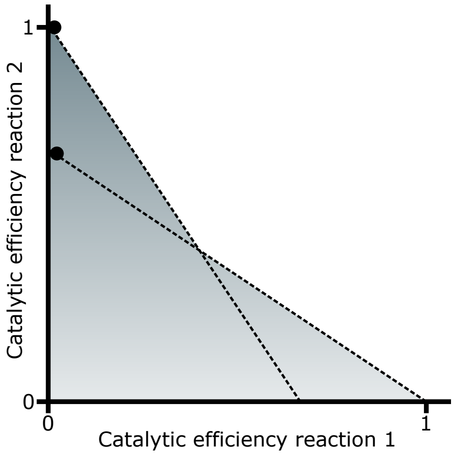

Figure S3: Using a 'divide and conquer' approach to find solutions in concave feasible spaces. The grey region denotes the feasible space of catalytic efficiencies (i.e. the region reachable by mutation) for a pair of reactions catalysed by the same enzyme. The constraints defining this space are shown as dashed lines, i.e. every combination of catalytic efficiencies beneath at least one of the lines but above a catalytic efficiency of zero corresponds to a possible enzyme variant. The feasible space is not convex, i.e. not all straight lines between points within the space remain within it. We model natural selection as an optimisation problem that maximizes fitness by increasing catalytic efficiency as far as the constraints allow. However, the optimisation algorithm we use does not work within a non-convex space. In order to solve this non-convex optimisation problem, we divide the feasible space in two, namely into the two areas beneath each of the dashed lines (the two constraints). The resulting two subspaces are now convex and optima can be found for each one of the subspaces. In this hypothetical example, fitness (colour gradient, darker colour indicates higher fitness) is maximised by increasing the catalytic efficiency of reaction two and eliminating the catalysis of reaction one. Solving the two sub-problems yields two enzyme variants (black circles), one of which has a higher activity for reaction two than the other and consequently has a higher fitness. This circle indicates the global maximum of this optimisation problem. Scales are arbitrary: Zero denotes no catalytic activity, one maximum activity.

constraint.

S1.6 Building a biophysical model of enzyme promiscuity

We aimed to generate the null distribution of enzyme promiscuity index purely from enzyme kinetics. That is, we wanted to see how this distribution looks like for a protein that can catalyze two reactions in the absence of selection for enzymatic efficiency (k_{cat}/K_M) or promiscuity index. To do that, we first needed to generate the distribution of enzymatic efficiencies of single point mutations for an enzymatic reaction. We assume that a catalytic reaction proceeds by a Michaelis-Menten mechanism,

$$\mathrm{E}+\mathrm{S} \underset{k_r}{\overset{k_f}{\rightleftarrows}} \mathrm{ES} \xrightarrow{k_{cat}} \mathrm{E}+\mathrm{P}$$

Here, E and S represent the enzyme and the substrate, ES is the enzyme-substrate complex, and P is the product. The rate parameters k_f, k_r, and k_{cat} represent the kinetic constants of the forward reaction to enzyme-substrate complex, the reverse reaction from enzyme-substrate complex to reactants, and the rate constant of the catalytic step, respectively. Figure S4a shows the free energy diagram of this reaction. In this scheme, the catalytic efficiency (k_{cat}/K_M) can be expressed as,

$$\frac{k_{cat}}{K_M} = \frac{k_{cat}}{\frac{k_{cat}+k_r}{k_f}} \tag{3}$$

Assuming that the kinetic rate constant of the catalytic step is much higher than that of the reverse reaction (i.e. $k_{cat} \gg k_r$), the enzymatic efficiency k_{cat}/K_M simlifies as

$$\frac{k_{cat}}{K_M} \approx k_f. \tag{4}$$

Equation 4 means that the enzymatic efficiency of an enzyme can be expressed in terms of the single kinetic constant of the forward reaction k_f. We used the Arrhenius equation to further expand equation 4,

$$x = \frac{k_{cat}}{K_M} \approx k_f \approx e^{-\frac{1}{k_B T}\Delta G^{\#}}. \tag{5}$$

Here, k_B is the Boltzmann constant, T is the temperature and $\Delta G^{\#}$ is the activation binding free energy of the substrate to the enzyme. Equation 5 is sufficient to generate the distributions of enzymatic efficiencies explored in figure 2 of the main text. The catalytic efficiency x, estimated from activation energy $\Delta G^{\#}$ can be inserted into the promiscuity index equation (main text, methods, equation 1, see also [1]) to estimate enzyme promiscuity.

S1.7 Ascertainment bias does not explain the empirical distribution of promiscuity

Here we investigate whether the empirical promiscuity distribution may be a consequence of ascertainment or measurement bias in the selection of substrates. Many of the substrates reported

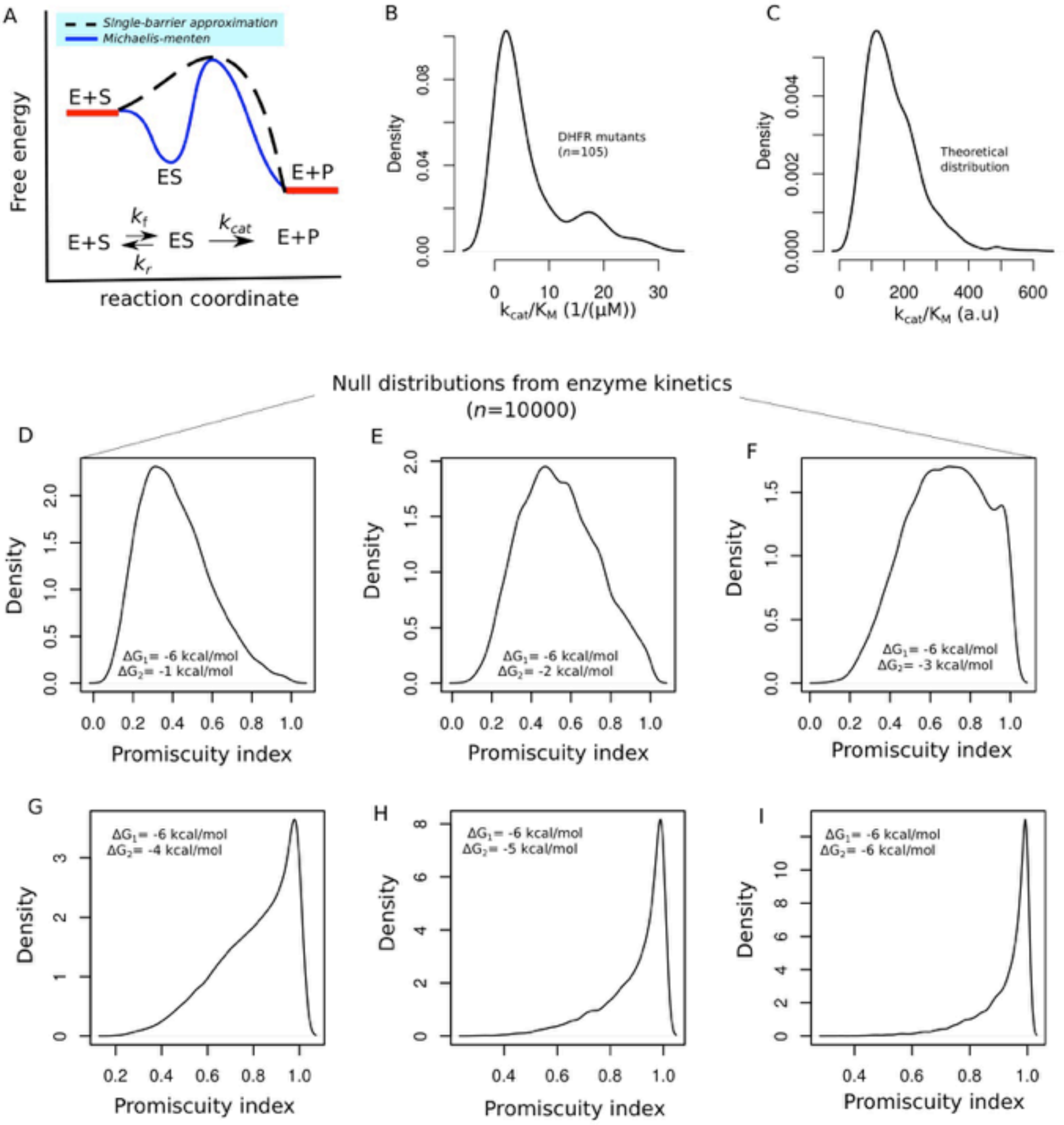

Figure S4: Enzyme kinetics help generates a null model for the expected distribution of enzyme promiscuity. A) The kinetic scheme and free energy diagram of an enzymatic reaction that follows the Michaelis-Menten kinetics (in blue), and an enzymatic reaction following a single energy barrier (dashed line). B) The distribution of enzymatic efficiency for 105 mutants of dihydrofolate reductase (DHFR) in reaction with dihydrofolate (DHF). C) The theoretical distribution of enzymatic efficiencies of $n = 10000$ mutants generated from enzyme kinetics (equation 5), with $\langle \Delta G^{\#} \rangle = -6$ kcal/mol and $\sigma_{\Delta G^{\#}} = 1$ kcal/mol. The null distributions of enzyme promiscuity for two reactions with D) $\Delta G^{\#}_{rxn1} = -6$ kcal/mol and $\Delta G^{\#}_{rxn2} = -1$ kcal/mol, E) $\Delta G^{\#}_{rxn1} = -6$ kcal/mol and $\Delta G^{\#}_{rxn2} = -2$ kcal/mol, F) $\Delta G^{\#}_{rxn1} = -6$ kcal/mol and $\Delta G^{\#}_{rxn2} = -3$ kcal/mol, G) $\Delta G^{\#}_{rxn1} = -6$ kcal/mol and $\Delta G^{\#}_{rxn2} = -4$ kcal/mol, H) $\Delta G^{\#}_{rxn1} = -6$ kcal/mol and $\Delta G^{\#}_{rxn2} = -5$ kcal/mol, I) $\Delta G^{\#}_{rxn1} = -6$ kcal/mol and $\Delta G^{\#}_{rxn2} = -6$ kcal/mol.

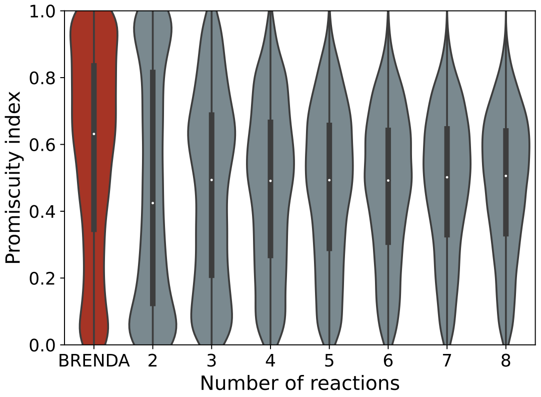

Figure S5: Samples from correlated multivariate log-normal distributions shift from a bimodal distribution of promiscuity index values to a unimodal distribution as the number of reactions per enzyme increases. White dots show the median promiscuity index, boxes contain the 25 and 75 percentiles, and vertical lines contain all promiscuity index values within 1.5 times the interquartile range. The violin plots are Gaussian kernel density estimates of the distributions of promiscuity index values. The distribution of promiscuity index values of empirical enzymes from BRENDA [7] is shown in red, the promiscuity index values of correlated random samples from log-normal bivariate or multivariate distributions with between three to eight dimensions in grey. The number of enzymes in the BRENDA dataset is 5028. Random samples comprise 5000 enzymes each.

for an enzyme are probably well-studied substrates with moderate to high catalytic efficiencies, which will cause the reported catalytic parameters for reactions with these substrates to be more similar to one another than if the enzyme's substrates had been sampled in an unbiased fashion. This hypothesis may explain why the catalytic efficiencies of substrates catalysed by the same enzyme are correlated.

We explored this hypothesis through correlated random sampling from bi- and multivariate distributions parametrised by the data from BRENDA (methods). This sampling suggests that measurement bias will only result in a bimodal distribution if few substrates are characterised per enzyme (figure S5). Specifically, only the simulated distribution of promiscuities with two substrates per enzyme is bimodal, though it is significantly different from the empirical distribution (two-sample Kolmogorov-Smirnov test, $D_{5000,5028} = 0.204$, $p = 1.16 \times 10^{-91}$). At higher numbers of substrates the promiscuity distribution quickly becomes unimodal (figure S5). The correlation between catalytic efficiencies for different reactions catalysed by the same enzyme is necessary to observe a bimodal distribution at all. If we assume that catalytic efficiencies for two reactions involving different substrates are not correlated, bimodality disappears altogether and the distribution becomes heavily biased towards low promiscuity (supplementary materials, figure S6). In sum, if sampling bias is the cause of the bimodality in the distribution of promiscuity, the discovery of new substrates for an enzyme will cause a shift in distribution of promiscuity towards unimodality. The empirical data is not consistent with this hypothesis, because 61 percent of the enzymes in the dataset have more than two known substrates, yet the promiscuity distribution is still bimodal.

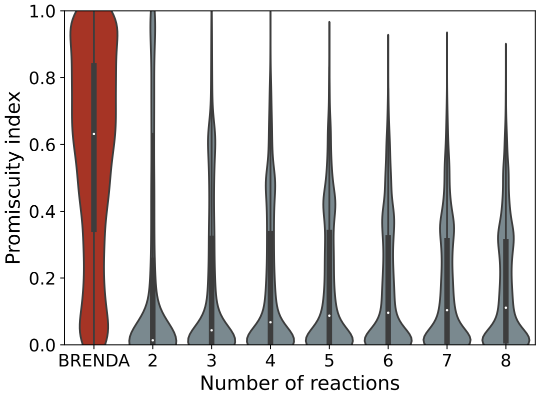

Figure S6: Samples from uncorrelated multivariate log-normal distributions create distributions of promiscuity indices biased towards low promiscuity index values, with the bias increasing as the number of reactions per enzyme increases (each reaction corresponds to one dimension). White dots show the median promiscuity index, boxes contain the 25 and 75 percentiles, and vertical lines contain all promiscuity index values within 1.5 times the interquartile range. The violin plots are Gaussian kernel density estimates of the distributions of promiscuity index values. The distribution of promiscuity indices of empirical enzymes from BRENDA [7] is shown in red, the promiscuity indices of uncorrelated random samples from log-normal bivariate or multivariate distributions with between three to eight dimensions in grey. The number of enzymes in the BRENDA dataset is 5028. Random samples comprise 5000 enzymes each.

S1.8 Specialisation requires strong trade-offs if selection favours catalysis from all duplicates for all reactions

We also investigated a scenario where fitness depends on the catalytic activities of all duplicates. In this scenario, the flux through a reaction depends on all duplicates that have catalytic activity for that reaction. Consequently, selection favours duplicates with overlapping activities, unless the gain in catalytic activity through specialisation for one reaction is larger than the loss of catalytic activity for other reactions combined. This scenario is relevant whenever duplication is selectively favoured as a means of increasing the expression of an enzyme [8, 9]. To assess whether duplication and specialization (rather than, for example, a simple increase in expression level) can be adaptive in this scenario, we compared the fitness of an organism expressing a promiscuous enzyme catalysing a given set of reactions with the fitness of an organism containing duplicates of this enzyme, each of which has specialised (as much as possible) for catalysing a single reaction only. The difference in fitness between these two organisms indicates in how far specialisation is favoured when duplication has already occurred.

We modelled this scenario as follows. First, we used the ‘specialist’ versions of the fitness function in equation 1 to model the specialisation of the duplicates by natural selection. We then took the catalytic efficiencies predicted by the optimisations performed on each duplicate and entered them into the ‘generalist’ version of the fitness function (equation 1 containing all n terms), because fitness depends on the catalysis of all n reactions.

When three reactions are highly synergistic, duplication and specialization have no effect on a cell’s ability to catalyse each reaction. As the pairwise relations between reactions become less synergistic and more antagonistic, the fitness effects of specialisation falls into two groups.

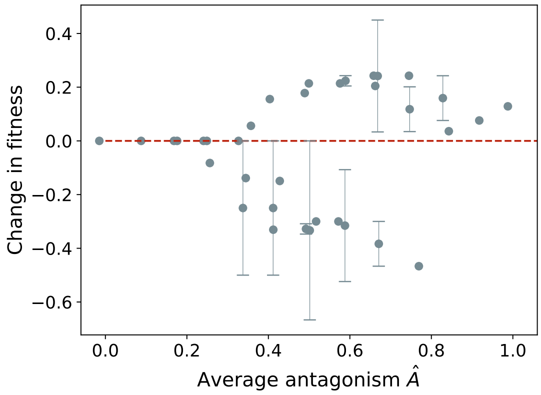

Figure S7: Duplication and specialization is only favoured if at least one pairwise relationship is strongly antagonistic. As the pairwise relationships between reactions become more antagonistic, selection either favours or disfavours specialisation. Fitness values range between zero and a maximum of one. We quantified to what extent different feasible space are synergistic or antagonistic by scoring the pairwise relationships in figure 3 with antagonism scores A (methods). The antagonism score A ranges from one (strongly antagonistic, figure 3a) to zero (strongly synergistic, figure 3e) in increments of 0.25. We characterise feasible spaces by the average antagonism score $\hat{A}$ of the pairwise relationships that constitute that feasible space. Consequently, $\hat{A} = 0$ denotes that all three pairwise relationships are strongly synergistic (figure 3e), while $\hat{A} = 1$ denotes that all pairwise relationships are strongly antagonistic (figure 3a). Data points have been slightly displaced along the horizontal axis to increase visibility. Error bars show variation in fitness after duplication due to variation in catalytic ability of the specialized enzymes for the reactions they have not been selected for. The red dashed line indicates no change in fitness, i.e., specialisation after duplication is neither beneficial nor deleterious but neutral. Fitness depends on the catalytic activities of all three enzyme variants that result from duplication and specialisation, and thus results are shown for each of the 35 feasible spaces.

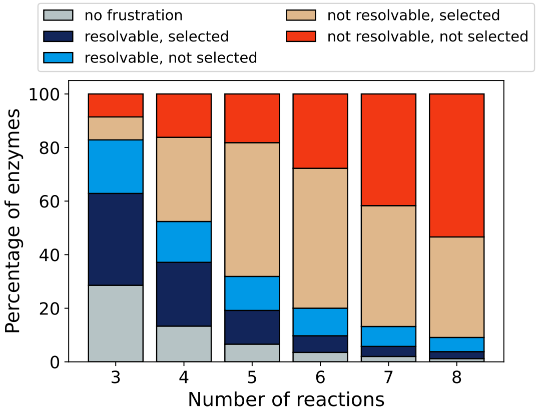

Figure S8: Irresolvable frustration becomes increasingly common as enzymes catalyse more reactions. Stacked bar plot of the percentage of enzymes whose feasible spaces fall into one of five classes. The first class contains enzyme variants that are not frustrated before or after duplication (grey). The second class contains enzyme variants that are frustrated before duplication, but where duplication can resolve frustration and different enzyme variants can specialise for different reactions if selection favours specialisation (dark blue). The third class contains feasible spaces where the resolution of frustration is possible but not selectively favoured (light blue). The fourth and fifth classes contain enzyme variants where duplication cannot entirely resolve frustration, with the fourth class containing spaces in which limited specialisation is favoured by selection (beige), and the fifth class enzyme variants where specialisation is not favoured by selection (orange). Data is based on 35, 210, 1001, 3876, 12650, and 35960 possible model enzymes with between 3 and 8 reactions, respectively.

In the first, specialisation is beneficial, and in the second specialization is detrimental. The first group always contains at least one pairwise relation that is strongly antagonistic, while the second group does not. This bifurcation in the fitness effects of specialisation is caused by the fact that fitness improves by maximizing flux through all reactions, and therefore fitness is maximized when an enzyme (or a set of duplicates) catalyses all three reactions with maximum efficiency. Consequently, unless two reactions are strongly antagonistic, the improvement in catalytic activity for one reaction gained through specialization is less than the loss in activity for the other two reactions. This result implies that some enzymes may be multifunctional not because greater specialization is not possible, but because specialization is not selectively favoured.

These observations suggest five classes of enzymes. The first class comprises enzymes that are not frustrated and highly promiscuous. The second comprises enzymes that are frustrated but where frustration can be resolved through duplication and specialization, and the third enzymes that are frustrated and where resolution is possible but not selectively favoured. The last two classes contain enzymes where frustration is not entirely resolvable due to interfering constraints, one class in which some specialisation is selected for, and another in which it is not. As the number of reactions per enzyme increases, interference between constraints becomes increasingly probable and the proportion of enzymes with irresolvable frustration (both with selection favouring some specialisation, and not) increases dramatically (figure S8).

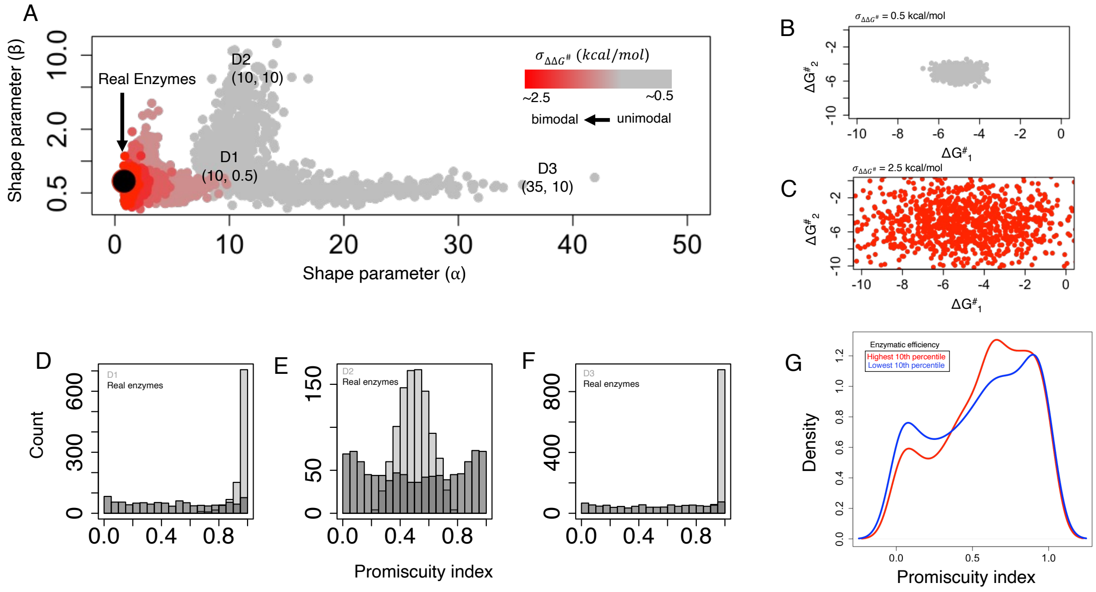

Figure S9: Mutations alone cannot fully explain the distribution of enzyme promiscuity in BRENDA database. A) The corresponding shape parameters obtained from fitting a beta distribution to real enzymes in BRENDA database (black circle), and modelled distributions from Equation 3. The theoretical distributions were generated by taking two activation free energies, labeled $\Delta G_1^{\#}$ and $\Delta G_2^{\#}$, from a Gaussian distribution with $\Delta G^{\#} = -5 \pm 2$ kcal/mol. We modeled the effect of mutations on activation free energy by generating distributions of $\Delta\Delta G^{\#}$ values centered on $\Delta G_1^{\#}$ and $\Delta G_2^{\#}$, with standard deviations ranging from 0.5 kcal/mol (in gray) to 2.5 kcal/mol (red). The three representative distributions D1, D2, and D3 plotted in D-F are shown here with their corresponding shape parameters. B, C) The activation free energy of the first reaction ($\Delta G_1^{\#}$) versus the activation free energy of the second reaction ($\Delta G_2^{\#}$) for $\Delta\Delta G^{\#}$ distributions with the standard deviation of 0.5 kcal/mol (panel B), and 2.5 kcal/mol (panel C). D, E, F) The histograms for three representative distributions of enzyme promiscuity index, D1 (panel D), D2 (panel E), and D3 (panel F) plotted with the histogram generated using the parameters of beta distribution that fit the distribution of real enzymes in BRENDA (alpha=beta=0.85). Each histogram was generated with 1000 data points. G) The distributions of enzyme promiscuity for real enzymes in the BRENDA database whose average enzymatic efficiencies are ranked in the highest (in red) and lowest (in blue) 10th percentile of the database.

S1.9 Single point mutations are not sufficient to explain bimodal distribution of promiscuity

To understand how evolution shapes the distribution of enzyme promiscuity, we first examine how mutations can alter this distribution. To do this in a biologically realistic way we take a biophysical approach and ask how mutations can change the enzyme promiscuity by affecting the activation free energy of enzymatic reactions. We chose to investigate an enzyme that is capable of catalysing two distinct reactions. Mutations occurring within this enzyme can increase or decrease the efficiency of either reaction. However, the distribution of changes in enzymatic efficiency resulting from these mutations is not well understood. Using Michaelis-Menten kinetics, we were able to derive a formula that estimates enzyme promiscuity I from the activation free energy of enzymatic reactions, $\Delta G^{\#}$ (see supplementary materials section S1.6 for details). The distribution of the activation free energy of an enzymatic reaction has been previously measured through

experimental and theoretical approaches for different enzymes. It follows a Gaussian distribution with a mean centered on -4 to -7 kcal/mol and a standard deviation of about 2 kcal/mol [10].

To determine the expected distribution of enzyme promiscuity resulting from single point mutations, without any specific constraint on enzymatic efficiency, we carried out a two-step calculation. In the first step, we selected two binding free energies from a Gaussian distribution with the mean of -5 kcal/mol and a standard deviation of 2 kcal/mol. These two free energies, labelled $\Delta G_1^{\#}$ and $\Delta G_2^{\#}$, correspond to the activation free energies of two enzymatic reactions of our desired enzyme. In the second step, we modeled the effect of mutations on activation free energy by generating distributions of $\Delta\Delta G^{\#}$ values. We generated two Gaussian distributions centered on $\Delta G_1^{\#}$ and $\Delta G_2^{\#}$, with different standard deviations ranging from 0.5 to 2.5 kcal/mol. We did that to account for the uncertainty of the variation of the activation free energy of enzymatic reactions by mutations. We then randomly selected 100 pairs of activation free energies from these distributions with replacement, and for each pair, we first converted these activation energies to catalytic efficiencies using equation 5, and then used equation 2 in the main text [1] to calculate the promiscuity index.

We repeated this two-step procedure 1000 times and obtained 1000 distributions for enzyme promiscuity that were obtained from various combinations of $\Delta G_1^{\#}$ and $\Delta G_2^{\#}$. We then fitted a beta distribution to all these distributions as well as the distribution of enzyme promiscuity of natural enzymes from the BRENDA database (as shown in figure S9). We selected the beta distribution for modeling these distributions because it is commonly used to represent the probability distribution of variables when the distribution type is unknown. This distribution involves two positive shape parameters, alpha and beta, whose combination determines the shape and skewness of the distribution. Using this approach, we asked whether mutations could cause a bimodal distribution of enzyme promiscuity.

Figure S9a illustrates that, for highly efficient enzymes ($\sigma_{\Delta\Delta G^{\#}} = 0.5$ kcal/mol), the distribution of the promiscuity index is unimodal at all combinations of $\Delta G_1^{\#}$ and $\Delta G_2^{\#}$ values (see the three representative distributions D1, D2, and D3 in figures S9d-f). We observed that the distribution of enzyme promiscuity index becomes bimodal only when the variation in the activation free energy substantially increases from $\sigma_{\Delta\Delta G^{\#}} = 0.5$ kcal to 2.5 kcal/mol, which is in contrast with our findings in real enzymes. Simply put, we get a bimodal distribution in our biophysical model when we consider both highly and lowly efficient enzymes while in real enzymes, bimodality exists even for highly efficient enzymes. As shown in figure S9f, the distribution of enzyme promiscuity remains bimodal for enzymes whose average efficiencies are ranked in the highest 10th percentile of the database. These results demonstrate that while mutations can lead to a diverse range of enzymatic efficiencies that exhibit a bimodal distribution, they cannot fully account for why enzymatic efficiencies are bimodal even at very high levels of efficiency. Therefore, we hypothesized that other mechanisms play a role in shaping the distribution of promiscuity index.

S1.10 Finding putative orthologs

For the 2039 enzymes with protein accession numbers, we used UniprotR [11] to download their amino acid sequences from Uniprot [12]. We then ran a BLAST search [13] in orthologR [14] of this database against itself to identify potential homologs, and filtered out any self-matches.

We then removed any putative paralogs by removing any entry where the BLAST hit came from the same species as the query sequence. We found 6,683 pairs of putative orthologs in this way. We then merged this dataset with the promiscuity data, where we discarded 81 pairs because the protein accession number had been replaced with a different identification during the download from Uniprot. For each pair of putative paralogs, we calculated the absolute difference in promiscuity. We then correlated this difference in promiscuity index with the percent sequence identity from the BLAST search, using the latter as a rough indication of the time since divergence.

References

1. Nath, A. & Atkins, W. M. A quantitative index of substrate promiscuity. *Biochemistry* **47,** 157–166 (2008).
2. Bar-Even, A. *et al.* The moderately efficient enzyme: evolutionary and physicochemical trends shaping enzyme parameters. *Biochemistry* **50,** 4402–4410 (2011).
3. Kacser, H. & Burns, J. A. The molecular basis of dominance. *Genetics* **97,** 639–666 (1981).
4. Kaltenbach, M. & Tokuriki, N. Dynamics and constraints of enzyme evolution. *J Exp Zool B Mol Dev Evo* **322,** 468–487 (2014).
5. Bershtein, S. *et al.* Protein homeostasis imposes a barrier on functional integration of horizontally transferred genes in bacteria. *PLOS Genet* **11,** e1005612 (2015).
6. Mahadevan, R. & Schilling, C. H. The effects of alternate optimal solutions in constraint-based genome-scale metabolic models. *Metab Eng* **5,** 264–276 (2003).
7. Jeske, L., Placzek, S., Schomburg, I., Chang, A. & Schomburg, D. BRENDA in 2019: a European ELIXIR core data resource. *Nucleic Acids Res* **47,** D542–D549 (2019).
8. Bergthorsson, U., Andersson, D. I. & Roth, J. R. Ohno's dilemma: evolution of new genes under continuous selection. *PNAS* **104,** 17004–17009 (2007).
9. Kondrashov, F. A. & Kondrashov, A. S. Role of selection in fixation of gene duplications. *J Theor Biol* **239,** 141–151 (2006).
10. Sousa, S. F. *et al.* Activation free energy, substrate binding free energy, and enzyme efficiency fall in a very narrow range of values for most enzymes. *ACS Catal* **10,** 8444–8453 (2020).
11. Soudy, M. *et al.* UniprotR: Retrieving and visualizing protein sequence and functional information from Universal Protein Resource (UniProt knowledgebase). *J Proteomics* **213,** 103613 (2020).
12. The UniProt Consortium. UniProt: the Universal Protein Knowledgebase in 2023. *Nucleic Acids Res* **51,** D523–D531 (2023).
13. Camacho, C. *et al.* BLAST+: architecture and applications. *BMC Bioinform* **10,** 421 (2009).

14. Drost, H.-G., Gabel, A., Grosse, I. & Quint, M. Evidence for Active Maintenance of Phylotranscriptomic Hourglass Patterns in Animal and Plant Embryogenesis. *Mol Biol Evol* **32,** 1221–1231 (2015).
